# Acidic microenvironment promotes pancreatic cancer cell survival via Akt phosphorylation-mediated suppression of apoptosis

**DOI:** 10.3389/fonc.2026.1889708

**Published:** 2026-09-22

**Authors:** Raito Yamamoto, Shigekuni Hosogi, Yuki Toda, Atsushi Shiozaki, Eishi Ashihara

**Affiliations:** 1 Laboratory of Clinical and Translational Physiology, Kyoto Pharmaceutical University, Kyoto, Japan; 2 Division of Digestive Surgery, Department of Surgery, Kyoto Prefectural University of Medicine, Kyoto, Japan

**Keywords:** acidic tumor microenvironment, AKT signaling, apoptosis, ERK1/2 signaling, G_0_/G_1_ cell cycle delay, pancreatic cancer

## Abstract

The microenvironment of many solid tumors is acidic (pH 6.5-6.9) due to hypoxia and enhanced glycolytic metabolism and has been implicated in tumor progression and therapeutic resistance. However, the mechanisms by which pancreatic cancer cells adapt to extracellular acidosis and maintain survival under acidic stress remain incompletely understood. This study aimed to investigate the effects of extracellular acidosis on pancreatic cancer cell proliferation, apoptosis, cell cycle regulation, and intracellular signaling. Human pancreatic cancer cell lines PK-59 and MIA-PaCa-2 were cultured under acidic conditions (pH 6.9). Proliferation, cell cycle phase distribution, apoptosis, and intracellular signaling were assessed using cell growth assays, WST-8 assays, flow cytometry, and western blotting. Pharmacological inhibition of Akt and ERK1/2 was performed to evaluate their functional roles under acidic conditions. Under acidic conditions, both cell lines appeared to show reduced proliferation, an increased G_0_/G_1_ population, decreased Cyclin D1 and CDK4/6 expression, and attenuated ERK1/2 phosphorylation. No apparent increase in apoptosis was observed. Acidic conditions also appeared to be associated with increased Akt phosphorylation and changes in apoptosis-related proteins, including decreased Bad and Bax and increased Bcl-xL. Pharmacological Akt inhibition under acidic conditions appeared to be accompanied by an increased apoptotic population together with increased Bad and Bax and decreased Bcl-xL expression. These observations may suggest an association between Akt signaling and survival-related cellular responses under acidic conditions; however, they do not establish a causal requirement for Akt signaling or a causal relationship between the observed protein changes and apoptosis. ERK1/2 inhibition appeared to be associated mainly with changes in cell cycle phase distribution and related protein expression, whereas its effects on apoptosis appeared to vary between the two cell lines. Collectively, these findings may be consistent with differential changes in proliferation-associated and survival-associated signaling under extracellular acidosis, although the functional relationships among these changes remain uncertain. The present findings were obtained exclusively *in vitro* and do not establish pathway-specific causality, therapeutic efficacy, or immediate clinical applicability. Further studies using complementary mechanistic approaches, appropriate *in vivo* models, and patient-derived systems will be required to determine the biological and clinical relevance of these observations.

## 1 Introduction

According to estimates from the International Agency for Research on Cancer (IARC), one in five individuals worldwide will develop cancer during their lifetime, and pancreatic cancer remains the sixth leading cause of cancer-related death globally (1). Unlike lung or colorectal cancer, its mortality rate has not declined substantially over recent decades, which may be attributable, at least in part, to the frequent diagnosis of pancreatic cancer at an advanced stage and the limited availability of effective therapeutic options (1). Consequently, pancreatic cancer continues to carry one of the poorest prognoses among solid malignancies, underscoring the need to better understand the biological mechanisms that may be associated with tumor persistence.

Rapid tumor growth and abnormal vascular architecture may be associated with insufficient perfusion within solid tumors and the development of hypoxic regions in the tumor microenvironment (TME) (2). Under hypoxic conditions, activation of hypoxia-inducible factor-1α (HIF-1α) has been reported to upregulate glucose transporters and glycolytic enzymes, which may be associated with increased reliance on glycolytic metabolism (3). This metabolic reprogramming, commonly referred to as the Warburg effect, may be accompanied by increased lactate and H^+^ production (3). To maintain intracellular pH homeostasis, cancer cells are thought to actively export excess H^+^ via several transporters, including Na^+^/H^+^ exchangers, monocarboxylate transporters, H^+^-ATPase, and Na^+^/HCO_3_^-^ cotransporters, as well as through carbonic anhydrase-mediated reactions (4–6). Consistent with these processes, the extracellular pH of solid tumors has been reported to range from 6.2 to 7.2, and acidic extracellular conditions have been observed in the TME (7).

Tumor acidosis has been reported to be associated with cancer cell survival and, in certain contexts, proliferation (8), as well as being associated with stem-like properties (9–11), epithelial-mesenchymal transition (EMT) with consequent invasion, migration, and metastasis (12–15), immune evasion (16, 17), and therapeutic resistance (18, 19). In pancreatic cancer, extracellular acidosis may be relevant to the tumor microenvironment, as this malignancy is characterized by extensive desmoplastic stroma and poor vascularization, features that may be associated with persistent hypoxia, nutrient deprivation, and extracellular acidosis (20–22). These microenvironmental characteristics may expose pancreatic cancer cells to sustained cellular stress and may be associated with adaptive cellular responses. In pancreatic cancer, oncogenic KRAS is known to activate multiple downstream signaling pathways, including PI3K/Akt and ERK1/2. These pathways have been associated with the regulation of cell survival and proliferation through downstream effectors such as Bcl-2 family proteins and G_1_-to-S phase regulatory proteins (23–25). Dysregulation of these signaling networks has been associated with pancreatic cancer aggressiveness, therapeutic resistance, and poor clinical outcomes (24, 26). Although extracellular acidosis has been reported to suppress the proliferation of many normal cell types, some cancer cells appear to retain the capacity to survive under acidic conditions, and altered cellular phenotypes have also been reported in this setting. Thus, acidic microenvironments may be associated with a range of cellular responses in cancer cells. The mechanisms by which pancreatic cancer cells respond to extracellular acidosis remain incompletely understood. In particular, it remains unclear how extracellular acidosis may be associated with changes in proliferative and survival-related signaling pathways in pancreatic cancer cells.

## 2 Materials and methods

### 2.1 Cell lines

PK-59 and MIA-PaCa-2 human pancreatic cancer cell lines were obtained from the Institute of Physical and Chemical Research (Saitama, Japan) and the American Type Culture Collection (ATCC; Manassas, VA, USA), respectively. PK-59 and MIA-PaCa-2 were selected as two independent pancreatic cancer cell lines to determine whether the effects of extracellular acidosis were reproducible across different pancreatic cancer models. These cell lines were established from a liver metastasis (27) and a primary pancreatic tumor (28), respectively, allowing us to evaluate whether the observed responses to extracellular acidosis were conserved across pancreatic cancer models established from different tumor sites. PK-59 cells were cultured in RPMI-1640 medium, whereas MIA-PaCa-2 cells were cultured in DMEM (4.5 g/L glucose). Both media were supplemented with 10% heat-inactivated fetal bovine serum and 1% penicillin-streptomycin. Cells were maintained at 37 °C in a humidified incubator with 5% CO_2_. Detailed procedures are described in the **Supplementary Materials and Methods**.

### 2.2 Culture conditions

Cells were cultured under Normal pH (pH 7.5) or Low pH (pH 6.9) conditions. The culture medium was adjusted using 1 M nitric acid (HNO_3_; Nacalai Tesque, Kyoto, Japan), and pH was confirmed prior to use. Detailed procedures are described in the **Supplementary Materials and Methods**.

### 2.3 Reagents

The Akt inhibitor MK-2206 and the ERK1/2 inhibitor SCH772984 were purchased from MedChemExpress LLC (Monmouth Junction, NJ, USA) and dissolved in dimethyl sulfoxide (DMSO; Nacalai Tesque, Kyoto, Japan).

### 2.4 Cell growth assay

Cell proliferation was assessed by direct cell counting using trypan blue solution. Cells were seeded in 24-well plates and pre-cultured for 24 h, followed by replacement with Normal pH or Low pH medium. Cells were counted every 24 h for up to 72 h. At 0 h, only viable cells were counted, whereas at 24, 48, and 72 h, both viable and dead cells were counted. Detailed procedures are described in the **Supplementary Materials and Methods**.

### 2.5 Cell proliferation assay

The inhibitory effects of Akt and ERK1/2 inhibition were evaluated using the WST-8 assay with Cell Counting Kit-8 reagent (Dojindo Laboratories, Kumamoto, Japan) according to the manufacturer’s protocol and as previously described (29, 30). Cells were seeded in 96-well plates and pre-cultured for 24 h, followed by replacement with Normal pH or Low pH medium and treatment with MK-2206 or SCH772984 for 72 h. Absorbance at 450 nm was measured using a microplate reader. Half-maximal inhibitory concentration (IC_50_) values were calculated using nonlinear regression analysis. For semi-logarithmic visualization, 0 M was replaced with a sufficiently small constant (1 × 10^–12^ M) to enable logarithmic transformation (31). Detailed procedures are described in the **Supplementary Materials and Methods**.

### 2.6 Phase-contrast imaging of cell morphology

Cell morphology was assessed by phase-contrast microscopy. Cells were seeded and pre-cultured for 24 h, followed by replacement with Normal pH or Low pH medium and culture for 72 h. For inhibitor studies, MK-2206 and SCH772984 were applied under both Normal pH and Low pH conditions. MK-2206 was used at the IC_50_ concentration determined under Normal pH conditions for each cell line (20 µM for PK-59 cells and 15 µM for MIA-PaCa-2 cells), whereas SCH772984 was used at the IC_50_ concentration determined under Normal pH conditions for each cell line (35 µM for PK-59 cells and 8 nM for MIA-PaCa-2 cells). After 72 h of treatment, representative phase-contrast images were acquired. Detailed procedures are described in the **Supplementary Materials and Methods**.

### 2.7 Cell cycle analysis

Cell cycle phase distribution was analyzed using propidium iodide (PI) staining followed by flow cytometry as previously described (29, 30). Cells were seeded and pre-cultured for 24 h, followed by replacement with Normal pH or Low pH medium and culture for 72 h. For inhibitor studies, MK-2206 and SCH772984 were applied under both Normal pH and Low pH conditions. MK-2206 was used at the IC_50_ concentration determined under Normal pH conditions for each cell line (20 µM for PK-59 cells and 15 µM for MIA-PaCa-2 cells), whereas SCH772984 was used at the IC_50_ concentration determined under Normal pH conditions for each cell line (35 µM for PK-59 cells and 8 nM for MIA-PaCa-2 cells). Detailed procedures are described in the **Supplementary Materials and Methods**.

### 2.8 Apoptosis analysis

Apoptosis was assessed by Annexin V/PI staining followed by flow cytometry according to the manufacturer’s protocol and as previously described (29, 30). Cells were seeded and pre-cultured for 24 h, followed by replacement with Normal pH or Low pH medium and culture for 72 h. For inhibitor studies, MK-2206 and SCH772984 were applied under both Normal pH and Low pH conditions. MK-2206 was used at the IC_50_ concentration determined under Normal pH conditions for each cell line (20 µM for PK-59 cells and 15 µM for MIA-PaCa-2 cells), whereas SCH772984 was used at the IC_50_ concentration determined under Normal pH conditions for each cell line (35 µM for PK-59 cells and 8 nM for MIA-PaCa-2 cells). Detailed procedures are described in the **Supplementary Materials and Methods**.

### 2.9 Western blotting

Protein expression was assessed by western blotting according to the manufacturer’s protocol and as previously described (29, 30). Cells were seeded and pre-cultured for 24 h, followed by replacement with Normal pH or Low pH medium and culture for 72 h. For inhibitor studies, MK-2206 and SCH772984 were applied under both Normal pH and Low pH conditions. MK-2206 was used at the IC_50_ concentration determined under Normal pH conditions for each cell line (20 µM for PK-59 cells and 15 µM for MIA-PaCa-2 cells), whereas SCH772984 was used at the IC_50_ concentration determined under Normal pH conditions for each cell line (35 µM for PK-59 cells and 8 nM for MIA-PaCa-2 cells). Protein bands were detected by chemiluminescence and quantified. β-actin was used as a loading control. Detailed procedures are described in the **Supplementary Materials and Methods**.

### 2.10 Statistical analysis

Statistical analyses were performed using GraphPad Prism 11. All experiments were performed in triplicate and repeated at least three times independently. Data are presented as the mean ± standard deviation (SD) or standard error of the mean (SEM). Comparisons between two groups were performed using an unpaired Student’s t-test. Comparisons among multiple groups were analyzed by one- or two-way analysis of variance (ANOVA), followed by Bonferroni’s multiple comparisons test. A p-value < 0.05 was considered statistically significant. Exact statistical tests and p-values are provided in the figure legends. *p < 0.05; **p < 0.01; ***p < 0.001; ns, not significant.

## 3 Results

### 3.1 Maintenance of extracellular pH and effects of acidic conditions on pancreatic cancer cell proliferation and apoptosis

The extracellular pH of both cell-free medium and culture supernatants was assessed to confirm the intended culture conditions. At the 72 h time point, the extracellular pH appeared to remain comparable to that measured at the time of medium replacement under both Normal and Low pH conditions (**Figures 1A, B**), which may be consistent with maintenance of the intended extracellular pH conditions during the culture period. The effects of acidic conditions on proliferation and survival in PK-59 and MIA-PaCa-2 cells were assessed using a cell growth assay. Acidic conditions appeared to be associated with lower growth ratios in PK-59 and MIA-PaCa-2 cells compared with Normal pH conditions (**Figure 1C**). However, cell proliferation was still observed, and the proportion of viable cells did not appear to differ markedly (**Figure 1D**). To examine factors that might be associated with the lower growth ratios, cell cycle analysis was performed. Cell cycle analysis appeared to indicate an increase in the G_0_/G_1_ population under Low pH conditions (**Figures 2A, B**; **Supplementary Figures 2A, B**). In apoptosis analysis, no apparent increase was observed in either early (Annexin V (+)/PI (-)) or late (Annexin V (+)/PI (+)) apoptotic populations (**Figures 2C, D**; **Supplementary Figures 2C, D**). Representative phase-contrast images appeared to show largely preserved cell morphology under acidic conditions, without obvious morphological alterations despite the lower growth ratios (**Supplementary Figures 1A–H**). Collectively, these observations may be consistent with an association between extracellular acidosis, reduced proliferative activity, and an increased G0/G1 population, without an apparent increase in apoptotic cell populations or marked morphological alterations.

**Figure 1:**
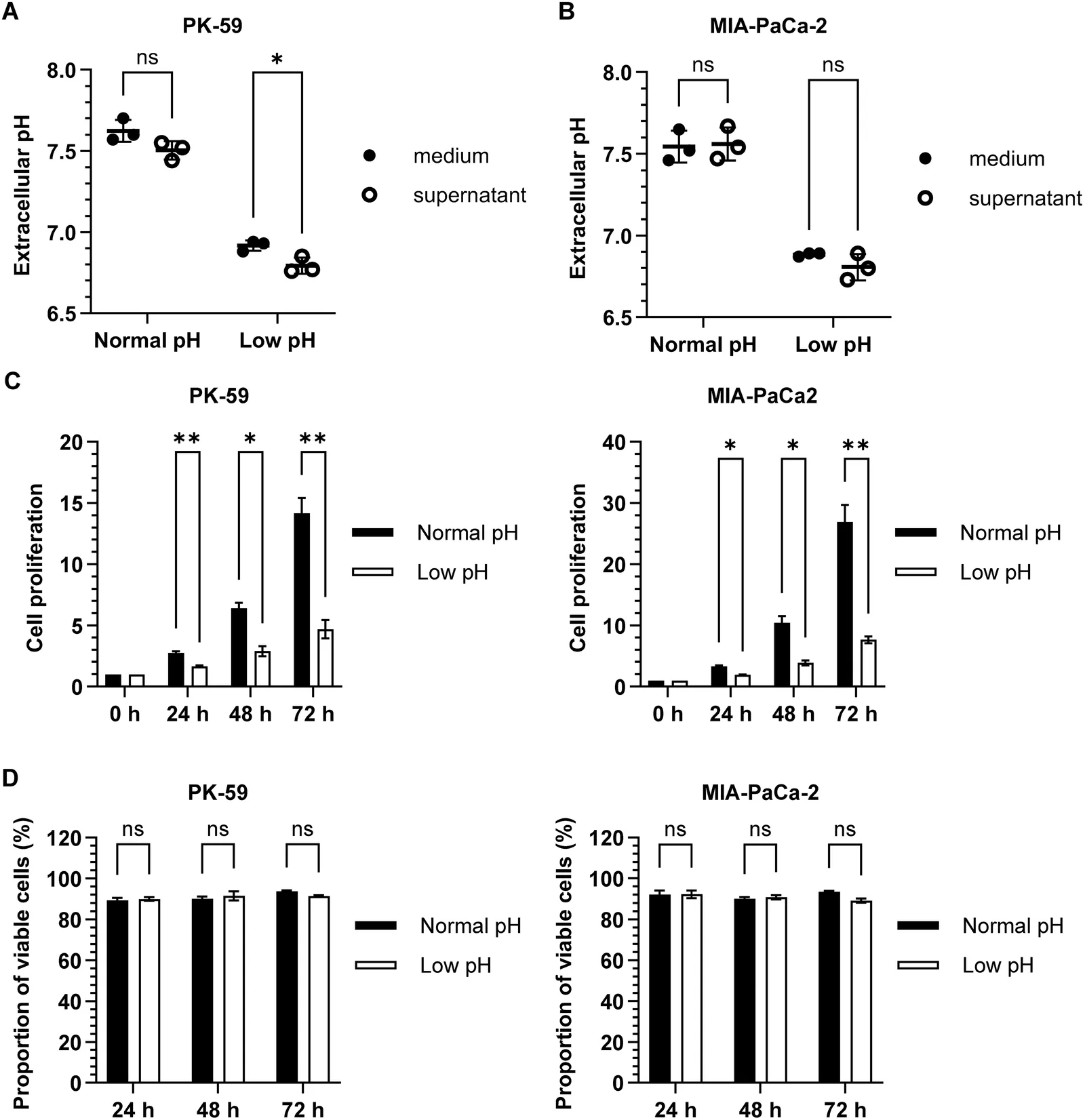
Maintenance of extracellular pH and effects of acidic conditions on cell proliferation and viability. **(A, B)** Extracellular pH of cell-free medium and culture supernatants collected at the 72 h time point under Normal pH and Low pH conditions in PK-59 **(A)** and MIA-PaCa-2 **(B)** cells. **(C)** Proliferation of PK-59 and MIA-PaCa-2 cells cultured under Normal pH or Low pH conditions. **(D)** Proportion of viable cells in PK-59 and MIA-PaCa-2 cells at 24, 48, and 72 h under Normal pH or Low pH conditions. **(A, B)** Data are presented as the mean ± standard deviation (SD) from three independent experiments, each performed in triplicate. **(C, D)** Data are presented as the mean ± standard error of the mean (SEM) from three independent experiments, each performed in triplicate. Statistical comparisons between two groups were performed using an unpaired Student’s t-test. *p < 0.05; **p < 0.01; ns, not significant.

**Figure 2:**
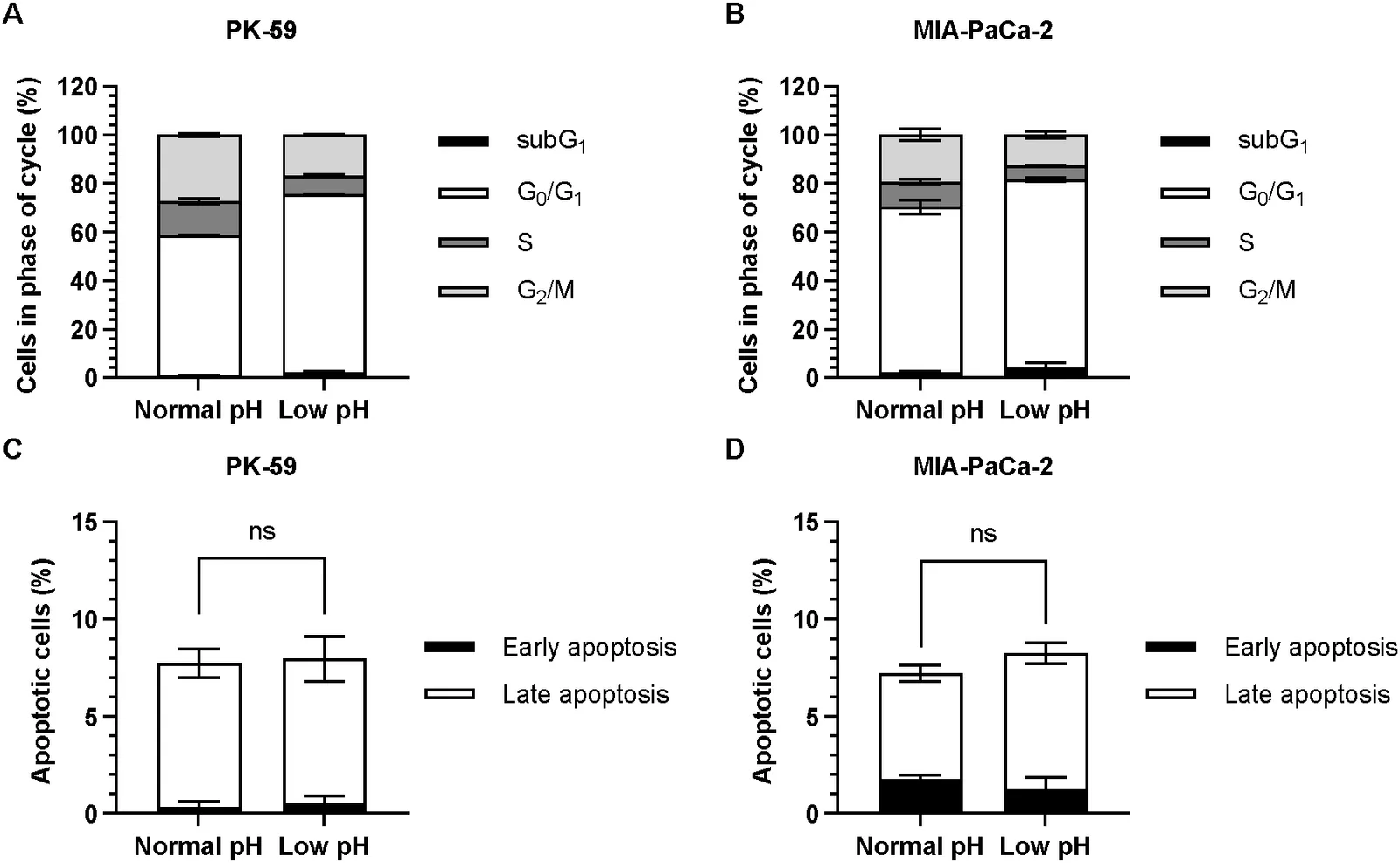
Effects of acidic conditions on cell cycle phase distribution and apoptosis. PK-59 and MIA-PaCa-2 cells were cultured for 72 h under Normal pH or Low pH conditions. **(A)** Cell cycle analysis of PK-59 cells. The G_0_/G_1_ population appeared to be increased under Low pH conditions (p < 0.001). **(B)** Cell cycle analysis of MIA-PaCa-2 cells. The G_0_/G_1_ population appeared to be increased under Low pH conditions (p = 0.0068). **(C)** Apoptosis analysis of PK-59 cells. **(D)** Apoptosis analysis of MIA-PaCa-2 cells. Data are presented as the mean ± SD from three independent experiments, each performed in triplicate. **(A, B)** Statistical comparisons between two groups were performed using an unpaired Student’s t-test. For clarity, statistical significance is described in the text rather than indicated in the graphs. **(C, D)** Statistical comparisons among multiple groups were performed using two-way analysis of variance (ANOVA), followed by Bonferroni’s multiple comparisons test. ns, not significant.

### 3.2 Effects of acidic conditions on G_0_/G_1_ cell cycle distribution and Cyclin D1/CDK4/6 expression

To examine factors potentially associated with the increased G_0_/G_1_ population under acidic conditions, proteins involved in the G_1_-to-S phase transition were analyzed by Western blotting. Western blot analysis appeared to show lower expression of Cyclin D1, CDK4, and CDK6 under Low pH conditions in both PK-59 and MIA-PaCa-2 cells (**Figures 3A–C, F–H**; **Supplementary Figures 3A–C, F–H**). In addition, phosphorylation of Rb at Ser807/811 appeared to be lower under Low pH conditions in MIA-PaCa-2 cells, whereas no apparent marked change was observed in PK-59 cells (**Figures 3D, I**; **Supplementary Figures 3D, I**). In contrast, p21 expression did not appear to differ markedly under acidic conditions in either cell line (**Figures 3E, J**; **Supplementary Figures 3E, J**). Collectively, these observations may be consistent with an association between the increased G_0_/G_1_ population and reduced Cyclin D1-CDK4/6 expression under acidic conditions. The apparent reduction in Rb phosphorylation in MIA-PaCa-2 cells may also be associated with this response in a cell line-dependent manner, whereas p21 did not appear to show a comparable change under the conditions examined (32).

**Figure 3:**
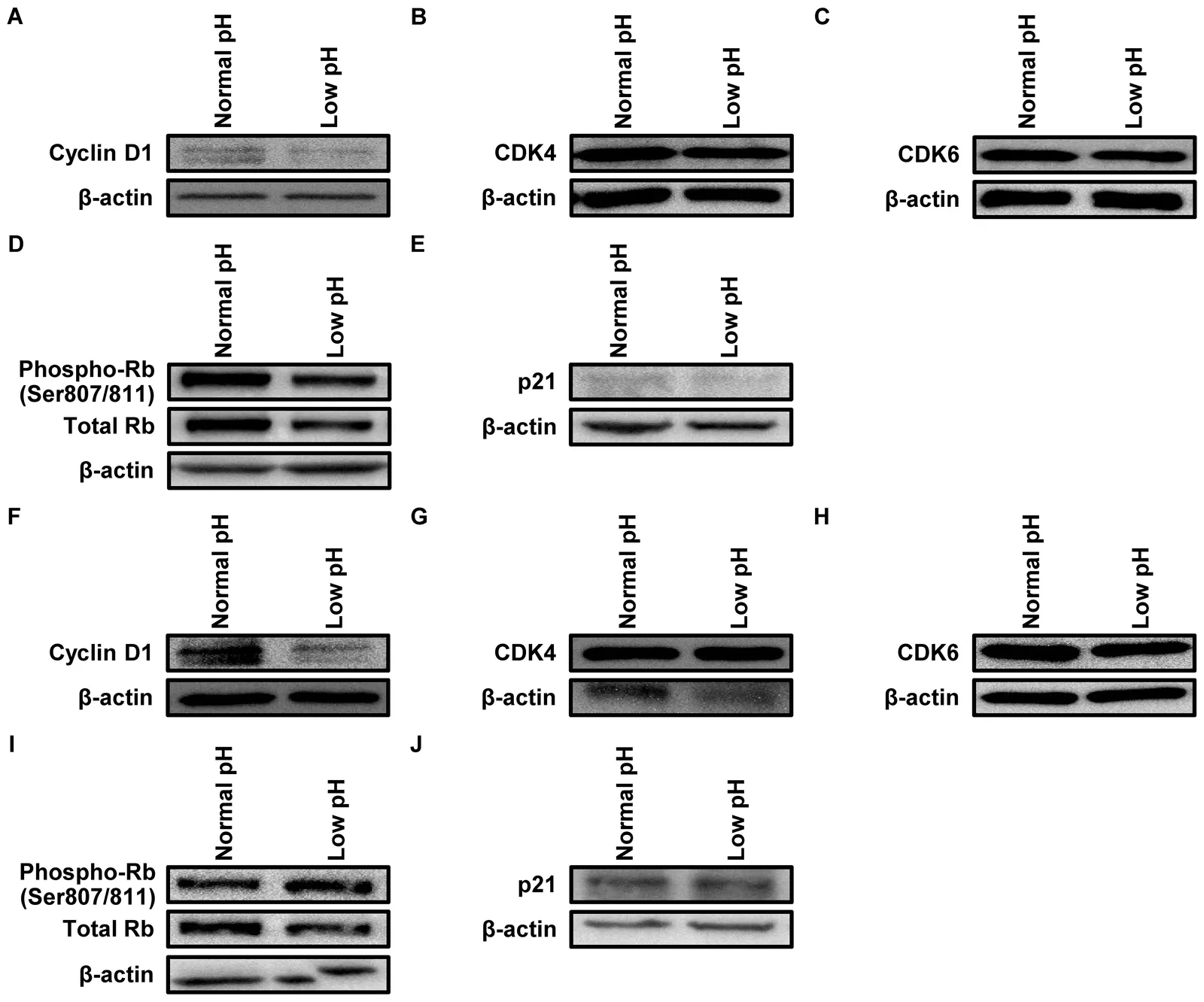
Effects of acidic conditions on the expression levels of proteins regulating the G_1_-to-S phase transition. Representative cropped immunoblot images of Cyclin D1, CDK4, CDK6, and p21 expression and Rb phosphorylation are shown for PK-59 and MIA-PaCa-2 cells cultured for 72 h under Normal pH or Low pH conditions. **(A, F)** Cyclin D1 expression in PK-59 and MIA-PaCa-2 cells, respectively. **(B, G)** CDK4 expression in PK-59 and MIA-PaCa-2 cells, respectively. **(C, H)** CDK6 expression in PK-59 and MIA-PaCa-2 cells, respectively. **(D, I)** Rb phosphorylation in PK-59 and MIA-PaCa-2 cells, respectively. Representative cropped immunoblot images of phospho-Rb, total Rb, and β-actin are shown. **(E, J)** p21 expression in PK-59 and MIA-PaCa-2 cells, respectively. All immunoblot images are representative of three independent experiments.

### 3.3 Effects of acidic conditions on apoptosis and Bad/Bax/Bcl-xL expression in pancreatic cancer cells

To examine factors potentially associated with the apoptotic response under acidic conditions, apoptosis-related proteins were analyzed by Western blotting. Bad and Bax appeared to show lower expression, whereas Bcl-xL appeared to show higher expression, under Low pH conditions (**Figures 4A–D**; **Supplementary Figures 4A–F**). These observations may be consistent with an association between extracellular acidosis and alterations in the expression of Bad, Bax, and Bcl-xL, although their functional contribution to the apoptotic response under these conditions remains uncertain (33, 34).

**Figure 4:**
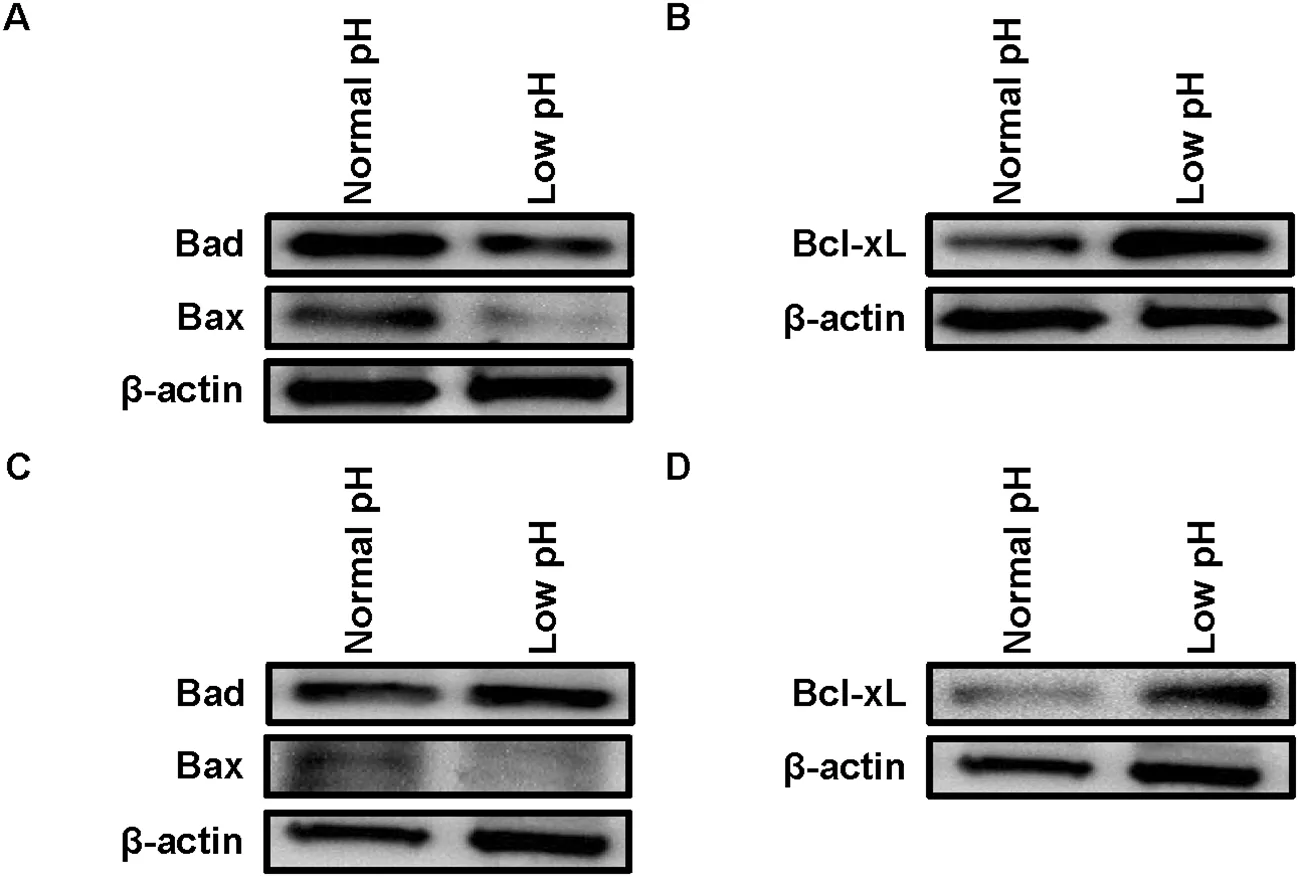
Effects of acidic conditions on the expression levels of proteins regulating apoptosis. Representative cropped immunoblot images of Bad, Bax, and Bcl-xL expression are shown for PK-59 and MIA-PaCa-2 cells cultured for 72 h under Normal pH or Low pH conditions. **(A, C)** Bad and Bax expression in PK-59 and MIA-PaCa-2 cells, respectively. **(B, D)** Bcl-xL expression in PK-59 and MIA-PaCa-2 cells, respectively. Representative cropped immunoblot images are shown. All immunoblot images are representative of three independent experiments.

### 3.4 Effects of acidic conditions on Akt and ERK1/2 phosphorylation in pancreatic cancer cells

Protein phosphorylation was analyzed by Western blotting to examine signaling changes potentially associated with cellular responses under acidic conditions. Akt phosphorylation appeared to be increased, whereas ERK1/2 phosphorylation appeared to be decreased under Low pH conditions (**Figures 5A–D**; **Supplementary Figures 5A–D**). These observations may be consistent with differential changes in Akt and ERK1/2 signaling under extracellular acidosis; however, the functional significance of these changes for cell survival and proliferation remains uncertain (35–37).

**Figure 5:**
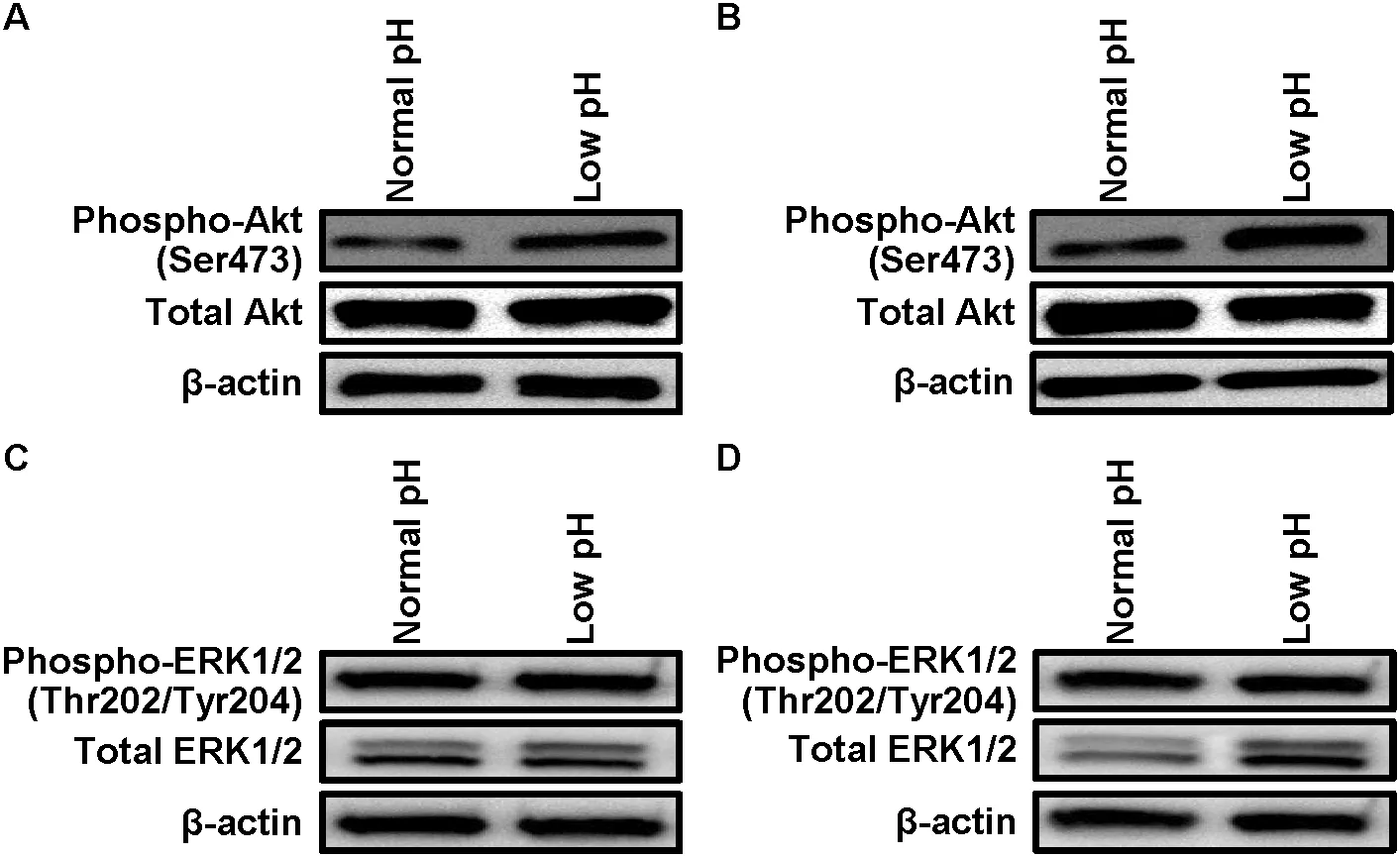
Effects of acidic conditions on Akt and ERK1/2 phosphorylation. PK-59 and MIA-PaCa-2 cells were cultured for 72 h under Normal pH or Low pH conditions. **(A, B)** Akt phosphorylation in PK-59 and MIA-PaCa-2 cells, respectively. **(C, D)** ERK1/2 phosphorylation in PK-59 and MIA-PaCa-2 cells, respectively. Representative cropped immunoblot images of phospho-Akt, total Akt, phospho-ERK1/2, total ERK1/2, and β-actin are shown. All immunoblot images are representative of three independent experiments.

### 3.5 Effects of Akt and ERK1/2 inhibition on pancreatic cancer cell proliferation under Normal and Low pH conditions

MK-2206, an Akt inhibitor, appeared to be associated with lower proliferation and lower IC_50_ values under Low pH conditions than under Normal pH conditions (**Figures 6A, B**; **Table 1**). In contrast, SCH772984, an ERK1/2 inhibitor, appeared to be associated with higher IC_50_ values under Low pH conditions (**Figures 6C, D**; **Table 2**). These observations may suggest that extracellular pH influences the response of pancreatic cancer cells to pharmacological inhibition of Akt and ERK1/2; however, they do not establish a specific dependence on either signaling pathway.

**Figure 6:**
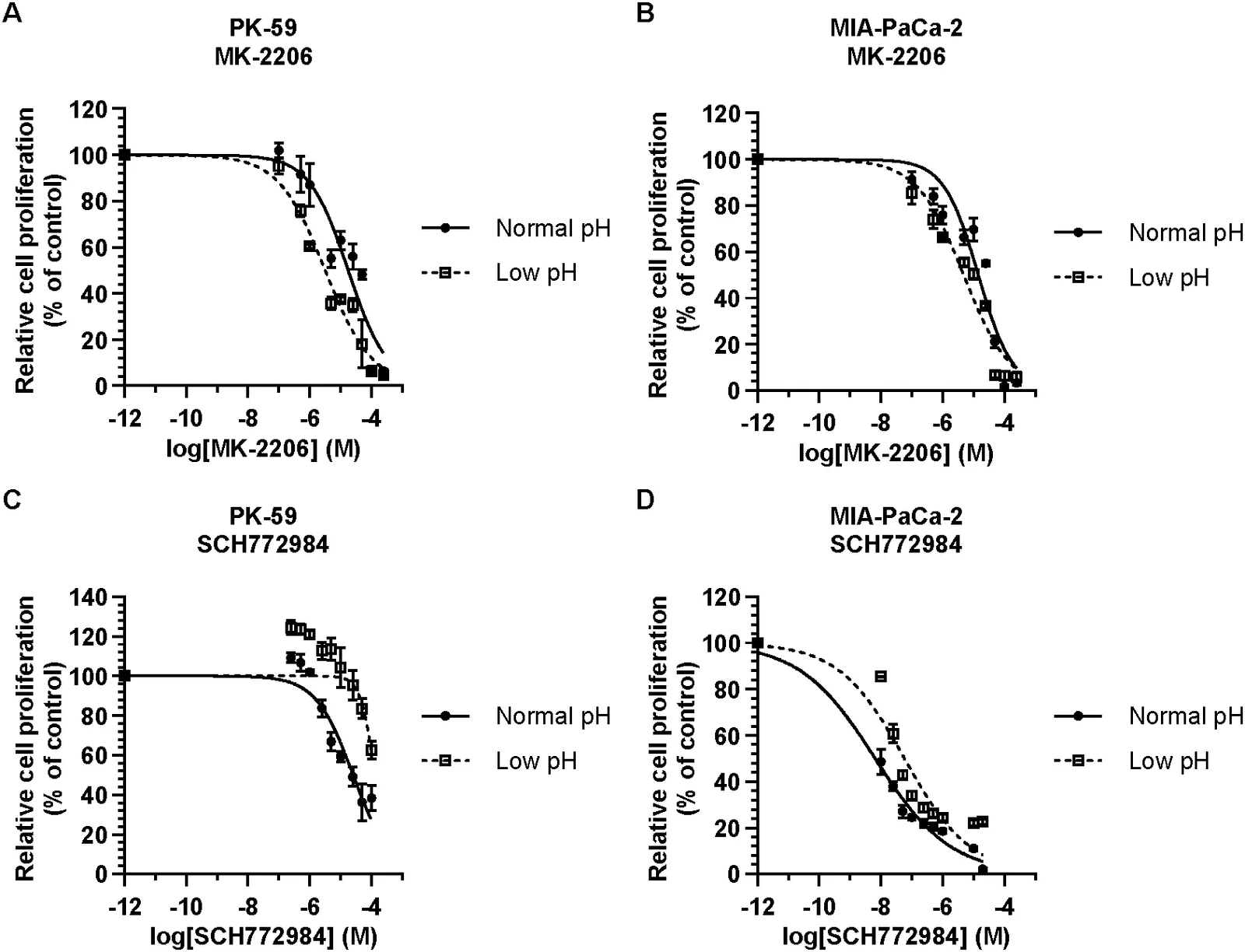
Effects of Akt and ERK1/2 inhibition on cell proliferation under acidic conditions. **(A, B)** Effects of the Akt inhibitor MK-2206 on the proliferation of PK-59 and MIA-PaCa-2 cells cultured for 72 h under Normal pH or Low pH conditions. **(C, D)** Effects of the ERK1/2 inhibitor SCH772984 on the proliferation of PK-59 and MIA-PaCa-2 cells cultured for 72 h under Normal pH or Low pH conditions. Data are presented as the mean ± SEM from three independent experiments, each performed in triplicate.

**Table 1:** IC_50_ values of the Akt inhibitor MK-2206 under Normal pH and Low pH conditions.

Cell lines	Normal pH	Low pH	p value (Normal pH vs Low pH)
PK-59 (Mean ± SD) (µM)	17.31 ± 4.84	3.26 ± 0.54	0.0075
MIA-PaCa-2 (Mean ± SD) (µM)	14.9 ± 5.44	4.87 ± 1.28	0.0359

Each experiment was performed in triplicate, and data represent the mean ± SD from three independent experiments. Statistical comparisons between two groups were conducted using an unpaired Student’s t-test.

**Table 2:** IC_50_ values of the ERK1/2 inhibitor SCH772984 under Normal pH and Low pH conditions.

Cell lines	Normal pH	Low pH	p value (Normal pH vs Low pH)
PK-59 (Mean ± SD) (µM)	33.74 ± 19.54	126.5 ± 14.58	0.0014
MIA-PaCa-2 (Mean ± SD) (nM)	7.37 ± 3.90	59.63 ± 18.14	0.0082

Each experiment was performed in triplicate, and data represent the mean ± SD from three independent experiments. Statistical comparisons between two groups were conducted using an unpaired Student’s t-test.

### 3.6 Effects of Akt inhibition on apoptosis and apoptosis-related protein expression in pancreatic cancer cells under acidic conditions

Akt phosphorylation following pharmacological Akt inhibition was analyzed by Western blotting. Akt inhibition appeared to be associated with reduced Akt phosphorylation under both pH conditions (**Supplementary Figures 6A–D**). Representative phase-contrast images appeared to show largely preserved cell morphology following Akt inhibition under both pH conditions, without obvious morphological alterations (**Supplementary Figures 7A–P**). Cell cycle analysis appeared to indicate an increased G_0_/G_1_ population following Akt inhibition under both Normal and Low pH conditions (**Figures 7A, B**; **Supplementary Figures 8A, B**). Western blotting appeared to show lower Cyclin D1 and CDK4/6 expression following Akt inhibition under Normal pH conditions, whereas under Low pH conditions their expression did not appear to differ markedly (**Figures 8A–C, E–H**; **Supplementary Figures 9A–C, E–H**). Rb phosphorylation appeared to be lower following Akt inhibition in MIA-PaCa-2 cells under Normal pH conditions, whereas no apparent marked change was observed under Low pH conditions. In PK-59 cells, Rb phosphorylation did not appear to change markedly following Akt inhibition under either pH condition (**Figures 8D, I**; **Supplementary Figures 9D, I**). Furthermore, p21 expression did not appear to differ markedly according to pH condition or Akt inhibition in either cell line (**Figures 8E, J**; **Supplementary Figures 9E, J**). Apoptosis analysis did not appear to indicate a marked increase in early or late apoptotic populations following Akt inhibition under Normal pH conditions (**Figures 9A, B**; **Supplementary Figures 10A, B**). In contrast, under Low pH conditions, pharmacological Akt inhibition appeared to be associated with an increased late apoptotic population. Western blotting also appeared to show higher Bad and Bax expression and lower Bcl-xL expression under Low pH conditions following Akt inhibition (**Figures 9C–F**; **Supplementary Figures 10C–H**), whereas no apparent marked changes in these proteins were observed under Normal pH conditions. Collectively, these observations may suggest that extracellular pH influences cellular responses to pharmacological Akt inhibition and that changes in Bad, Bax, and Bcl-xL expression occur concurrently with the increased apoptotic response under acidic conditions. However, these findings do not establish that Akt signaling is required for cell survival or that the observed changes in apoptosis-related proteins are causally responsible for the apoptotic response.

**Figure 7:**
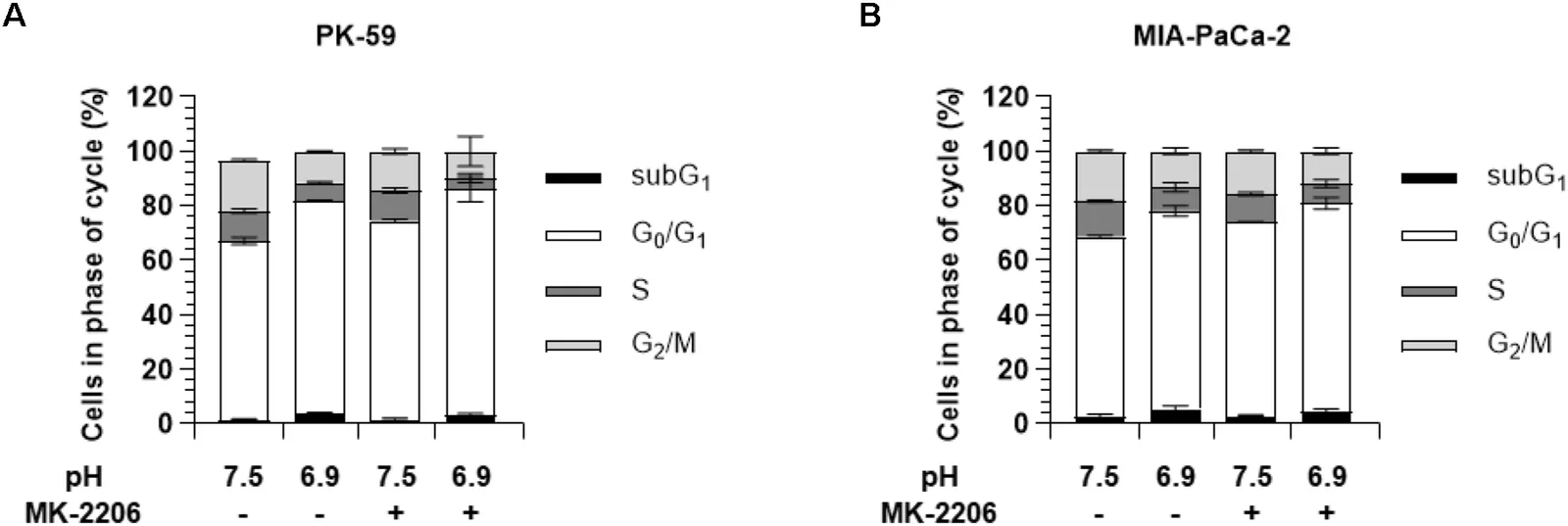
Effects of Akt inhibition on cell cycle phase distribution. Cell cycle analysis of PK-59 and MIA-PaCa-2 cells cultured for 72 h with or without MK-2206 under Normal pH or Low pH conditions. **(A)** The G_0_/G_1_ population appeared to be increased by MK-2206 under both Normal pH (p < 0.001) and Low pH conditions (p < 0.01). Under MK-2206 treatment, the G_0_/G_1_ population appeared to be higher under Low pH than under Normal pH conditions (p < 0.001). **(B)** The G_0_/G_1_ population appeared to be increased by MK-2206 under both Normal pH (p < 0.001) and Low pH conditions (p < 0.01). Under MK-2206 treatment, the G_0_/G_1_ population appeared to be higher under Low pH than under Normal pH conditions (p < 0.001). Statistical comparisons among multiple groups were performed using two-way ANOVA, followed by Bonferroni’s multiple comparisons test. For clarity, statistical significance is described in the text rather than indicated in the graphs.

**Figure 8:**
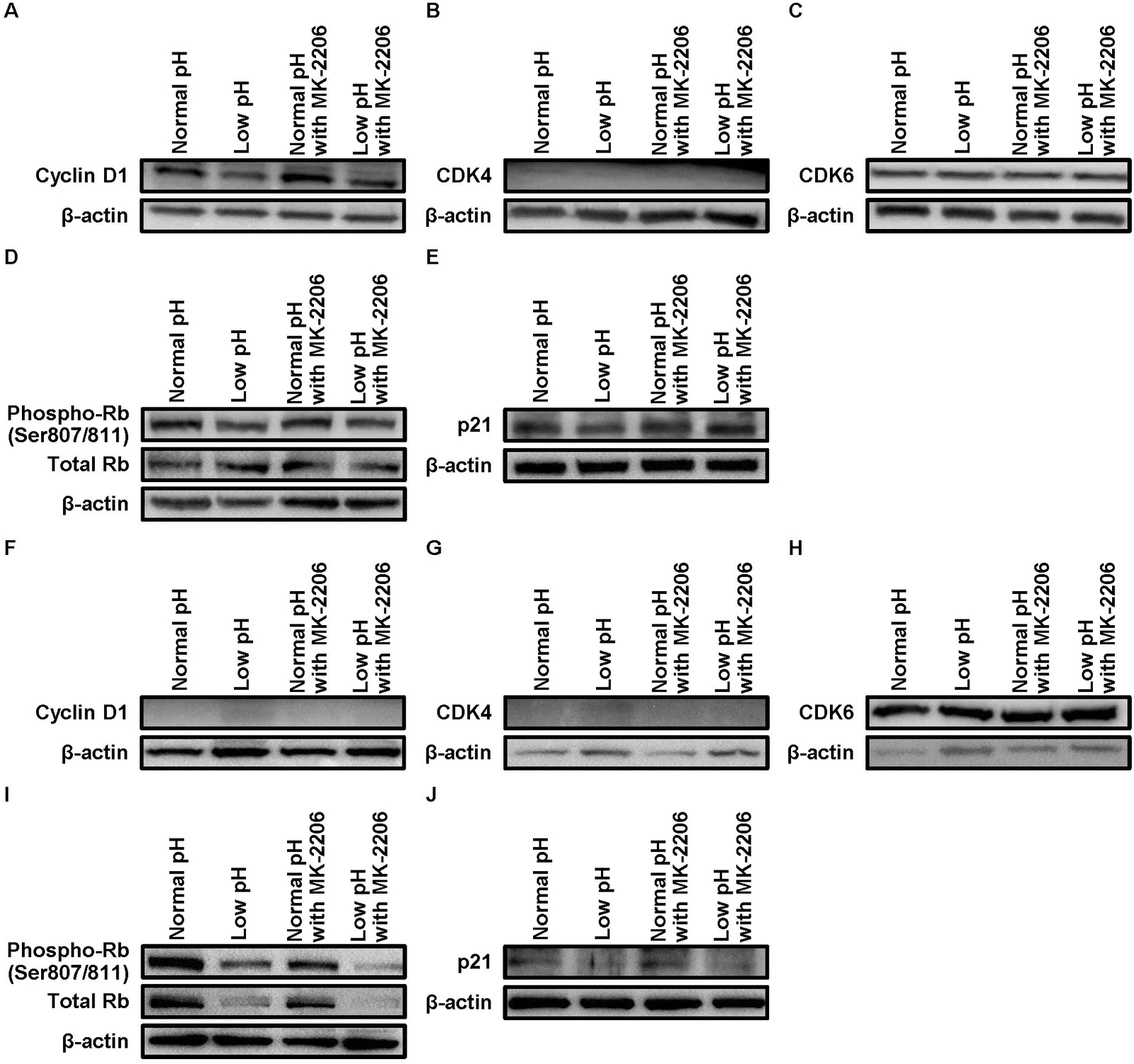
Effects of Akt inhibition on the expression levels of proteins regulating the G_1_-to-S phase transition. Representative cropped immunoblot images of Cyclin D1, CDK4, CDK6, and p21 expression and Rb phosphorylation are shown for PK-59 and MIA-PaCa-2 cells cultured for 24 h with or without MK-2206 under Normal pH or Low pH conditions. **(A, F)** Cyclin D1 expression in PK-59 and MIA-PaCa-2 cells, respectively. **(B, G)** CDK4 expression in PK-59 and MIA-PaCa-2 cells, respectively. **(C, H)** CDK6 expression in PK-59 and MIA-PaCa-2 cells, respectively. **(D, I)** Rb phosphorylation in PK-59 and MIA-PaCa-2 cells, respectively. Representative cropped immunoblot images of phospho-Rb, total Rb, and β-actin are shown. **(E, J)** p21 expression in PK-59 and MIA-PaCa-2 cells, respectively. All immunoblot images are representative of three independent experiments.

**Figure 9:**
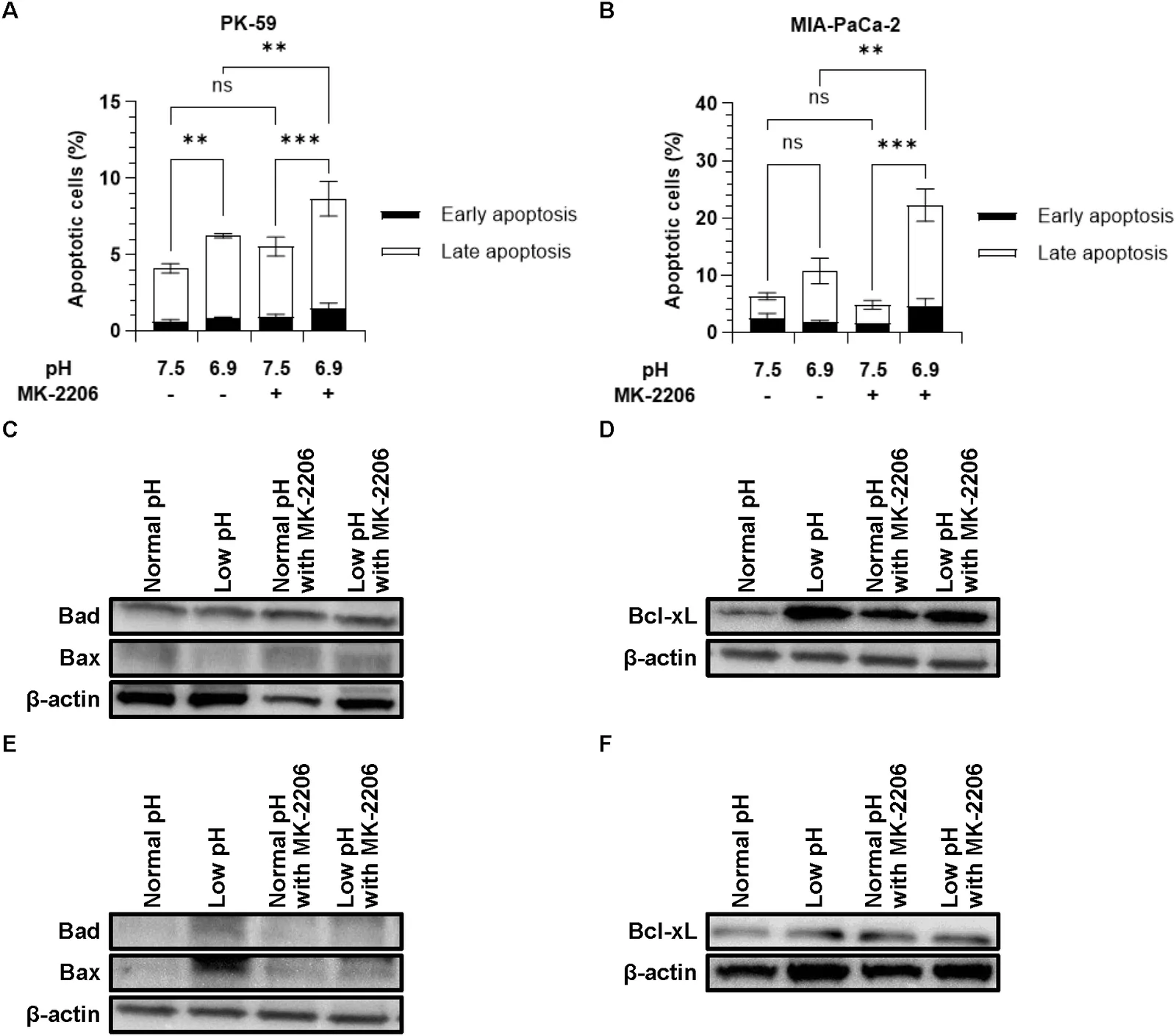
Effects of Akt inhibition on apoptosis and the expression levels of proteins regulating apoptosis. **(A, B)** Apoptosis analysis of PK-59 and MIA-PaCa-2 cells cultured for 72 h with or without MK-2206 under Normal pH or Low pH conditions. Statistical comparisons among multiple groups were performed using two-way ANOVA, followed by Bonferroni’s multiple comparisons test. **p < 0.01; ***p < 0.001; ns, not significant. **(C–F)** Representative cropped immunoblot images of Bad, Bax, and Bcl-xL expression are shown for PK-59 and MIA-PaCa-2 cells cultured for 24 h with or without MK-2206 under Normal pH or Low pH conditions. **(C, E)** Bad and Bax expression in PK-59 and MIA-PaCa-2 cells, respectively. **(D, F)** Bcl-xL expression in PK-59 and MIA-PaCa-2 cells, respectively. All immunoblot images are representative of three independent experiments.

### 3.7 Effects of ERK1/2 inhibition on G_0_/G_1_ cell cycle distribution, Cyclin D1/CDK4/6 and Bcl-xL expression, and apoptosis

ERK1/2 phosphorylation in PK-59 and MIA-PaCa-2 cells appeared to be reduced following SCH772984 treatment under both Normal and Low pH conditions (**Supplementary Figures 11A–D**). Representative phase-contrast images appeared to show modest morphological alterations in PK-59 cells following ERK1/2 inhibition, including an increased proportion of rounded and detached cells, whereas MIA-PaCa-2 cells did not appear to show obvious morphological changes under either pH condition (**Supplementary Figures 12A–P**). Cell cycle analysis appeared to indicate an increased G_0_/G_1_ population under Normal pH conditions in both cell lines, whereas under Low pH conditions this increase appeared to be observed only in MIA-PaCa-2 cells (**Figures 10A, B**; **Supplementary Figures 13A, B**). Western blotting appeared to show lower Cyclin D1 and CDK4/6 expression following ERK1/2 inhibition under both pH conditions (**Figures 11A–C, F–H**; **Supplementary Figures 14A–C, F–H**). Phosphorylated Rb did not appear to change markedly in PK-59 cells but appeared to be lower in MIA-PaCa-2 cells under both pH conditions (**Figures 11D, I**; **Supplementary Figures 14D, I**). In contrast, p21 expression did not appear to differ markedly in either cell line under either pH condition (**Figures 11E, J**; **Supplementary Figures 14E, J**). In PK-59 cells, apoptosis analysis appeared to indicate increases in early or late apoptotic populations under both pH conditions following ERK1/2 inhibition (**Figure 12A**; **Supplementary Figure 15A**). In contrast, no apparent marked increase in apoptosis was observed in MIA-PaCa-2 cells (**Figure 12B**; **Supplementary Figure 15B**). Western blotting appeared to show lower Bcl-xL expression in PK-59 cells. In MIA-PaCa-2 cells, Bcl-xL also appeared to be lower, whereas Bad and Bax did not show consistent increases (**Figures 12C–F**; **Supplementary Figures 15C–H**). Collectively, these observations may be consistent with an association between pharmacological ERK1/2 inhibition, changes in cell cycle phase distribution, and altered Cyclin D1/CDK4/6 expression. However, these findings do not establish that the observed changes in Cyclin D1/CDK4/6 are causally responsible for the changes in cell cycle distribution. The apoptotic response to ERK1/2 inhibition also appeared to differ between the two cell lines, and the functional significance of the accompanying changes in Bcl-xL expression remains uncertain.

**Figure 10:**
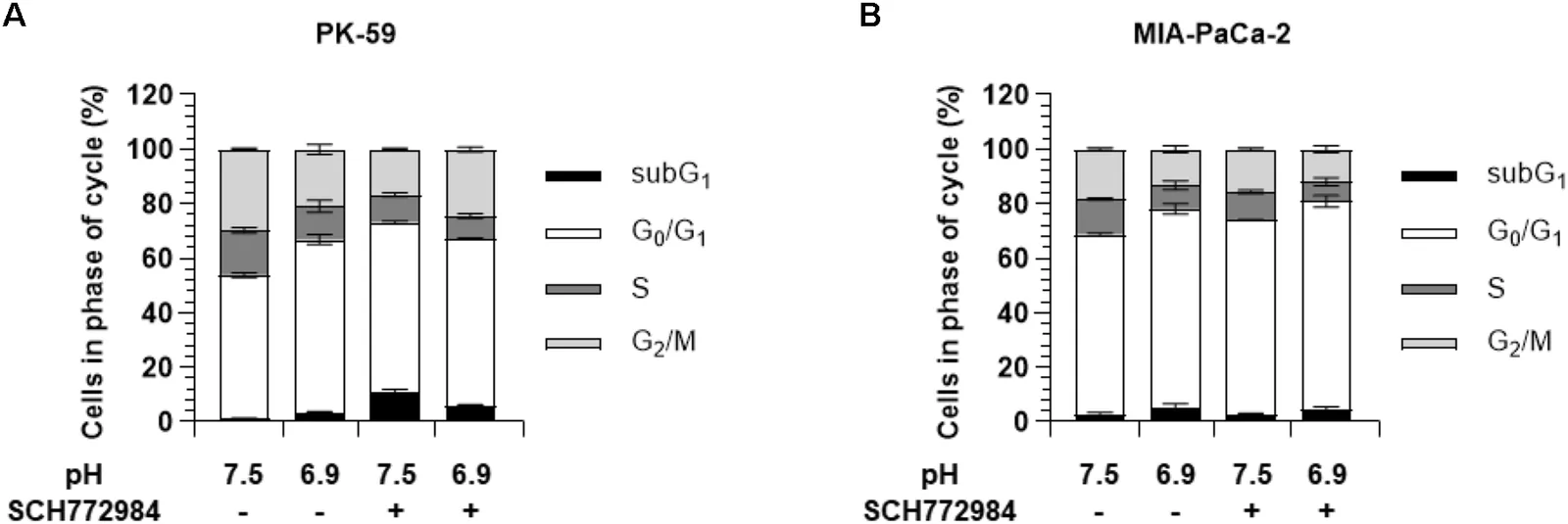
Effects of ERK1/2 inhibition on cell cycle phase distribution. Cell cycle analysis of PK-59 and MIA-PaCa-2 cells cultured for 72 h with or without SCH772984 under Normal pH or Low pH conditions. **(A)** In PK-59 cells, the G_0_/G_1_ population appeared to be increased by SCH772984 under Normal pH conditions (p < 0.001), whereas no apparent increase was observed under Low pH conditions. **(B)** In MIA-PaCa-2 cells, the G_0_/G_1_ population appeared to be increased by SCH772984 under both Normal pH (p < 0.001) and Low pH conditions (p < 0.01). Under SCH772984 treatment, the G_0_/G_1_ population appeared to be higher under Low pH than under Normal pH conditions (p < 0.001). Statistical comparisons among multiple groups were performed using two-way ANOVA, followed by Bonferroni’s multiple comparisons test. For clarity, statistical significance is described in the text rather than indicated in the graphs.

**Figure 11:**
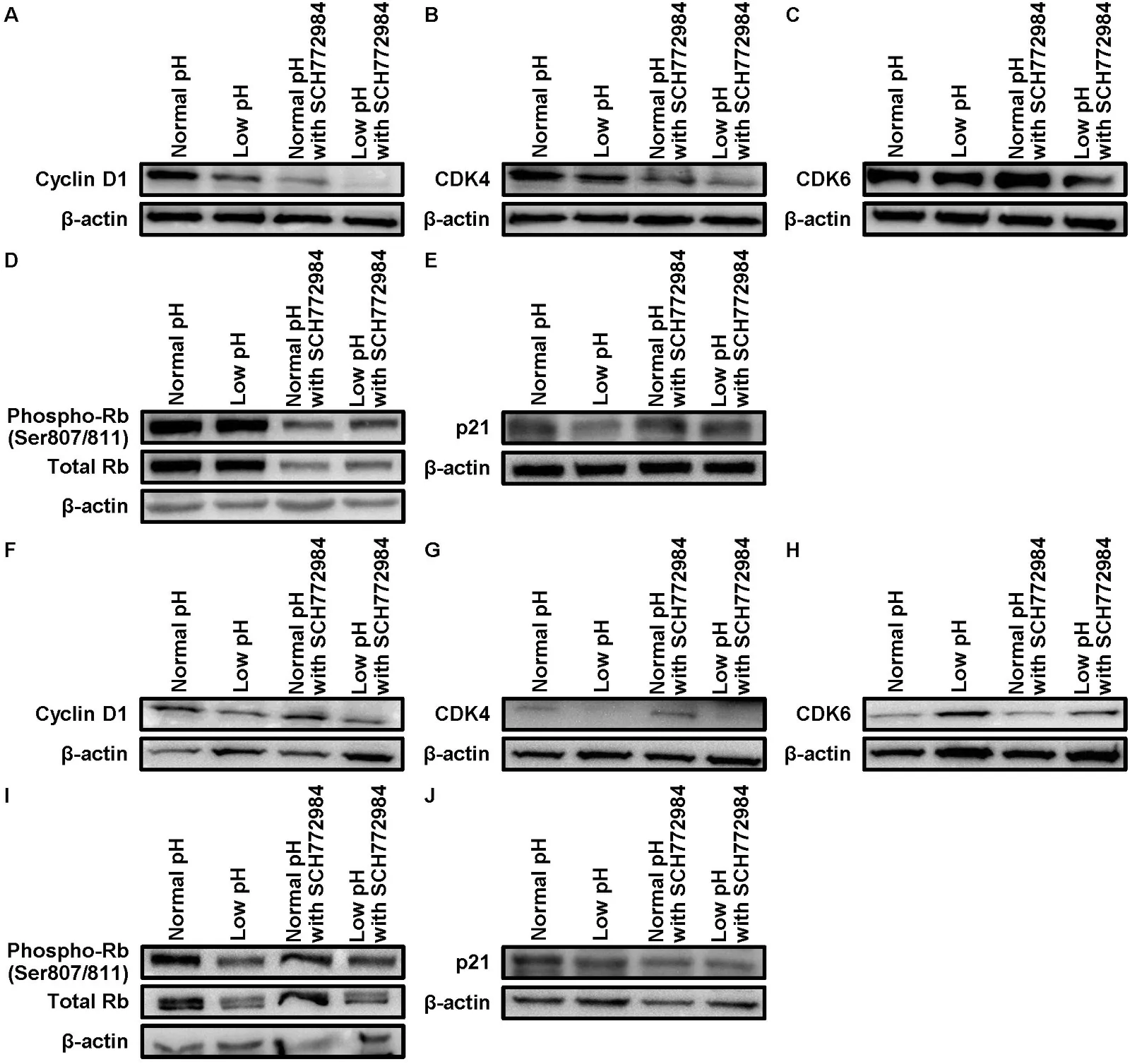
Effects of ERK1/2 inhibition on the expression levels of proteins regulating the G_1_-to-S phase transition. Representative cropped immunoblot images of Cyclin D1, CDK4, CDK6, and p21 expression and Rb phosphorylation are shown for PK-59 and MIA-PaCa-2 cells cultured for 24 h with or without SCH772984 under Normal pH or Low pH conditions. **(A, F)** Cyclin D1 expression in PK-59 and MIA-PaCa-2 cells, respectively. **(B, G)** CDK4 expression in PK-59 and MIA-PaCa-2 cells, respectively. **(C, H)** CDK6 expression in PK-59 and MIA-PaCa-2 cells, respectively. **(D, I)** Rb phosphorylation in PK-59 and MIA-PaCa-2 cells, respectively. Representative cropped immunoblot images of phospho-Rb, total Rb, and β-actin are shown. **(E, J)** p21 expression in PK-59 and MIA-PaCa-2 cells, respectively. All immunoblot images are representative of three independent experiments.

**Figure 12:**
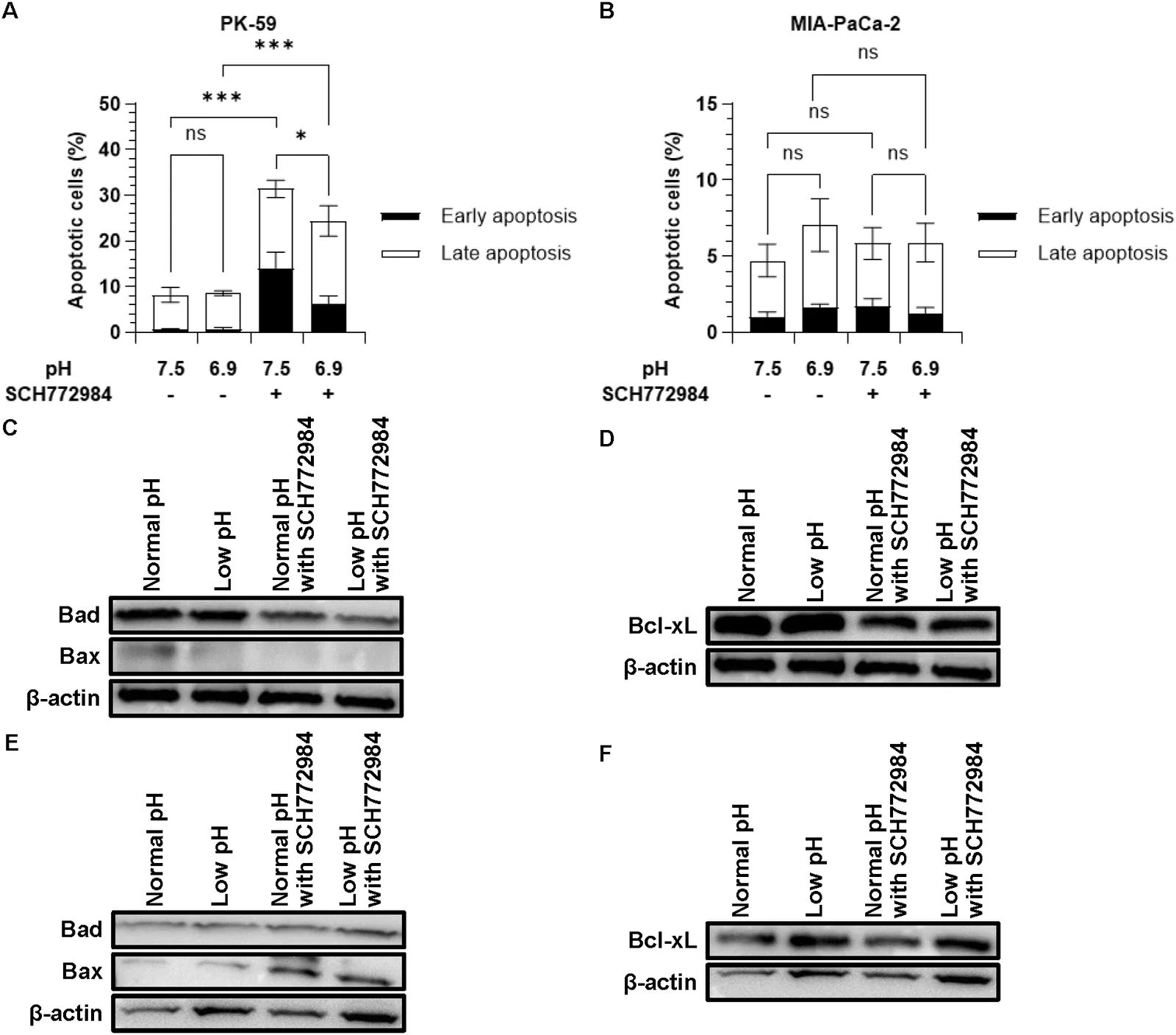
Effects of ERK1/2 inhibition on apoptosis and the expression levels of proteins regulating apoptosis. **(A, B)** Apoptosis analysis of PK-59 and MIA-PaCa-2 cells cultured for 72 h with or without SCH772984 under Normal pH or Low pH conditions. Statistical comparisons among multiple groups were performed using two-way ANOVA, followed by Bonferroni’s multiple comparisons test. *p < 0.05; ***p < 0.001; ns, not significant. **(C–F)** Representative cropped immunoblot images of Bad, Bax, and Bcl-xL expression are shown for PK-59 and MIA-PaCa-2 cells cultured for 24 h with or without SCH772984 under Normal pH or Low pH conditions. **(C, E)** Bad and Bax expression in PK-59 and MIA-PaCa-2 cells, respectively. **(D, F)** Bcl-xL expression in PK-59 and MIA-PaCa-2 cells, respectively. All immunoblot images are representative of three independent experiments.

## 4 Discussion

In the present study, we observed that extracellular acidosis appeared to be associated with reduced pancreatic cancer cell proliferation without an apparent increase in apoptosis. Under Low pH conditions, PK-59 and MIA-PaCa-2 cells appeared to show lower growth rates together with an increased G_0_/G_1_ population. Apoptotic cell populations did not appear to increase markedly under these conditions. Collectively, these observations may be consistent with an association between extracellular acidosis, reduced proliferative activity, and maintenance of cell viability; however, the mechanisms underlying these responses remain uncertain.

TME is frequently characterized by extracellular acidosis resulting from hypoxia and enhanced glycolytic metabolism. Acidosis has been implicated in promoting cancer cell survival (8), stem-like properties (9–11), and therapeutic resistance (18, 19), as well as in adaptive cellular responses to environmental stress. Acidic microenvironments have also been associated with metabolic adaptation, phenotypic plasticity, and the acquisition of stem cell-like features. However, the mechanisms by which pancreatic cancer cells respond to acidic conditions remain incompletely understood. The present observations may suggest that extracellular acidosis is accompanied by changes in both proliferation-associated and survival-associated cellular responses, although the functional relationships among these changes remain to be determined.

The PI3K/Akt and ERK1/2 pathways are well-recognized downstream effectors of oncogenic KRAS and have been implicated in pancreatic cancer cell survival and proliferation. In the present study, the increased G_0_/G_1_ population was accompanied by apparently lower Cyclin D1 and CDK4/6 expression and attenuated ERK1/2 phosphorylation. Pharmacological ERK1/2 inhibition also appeared to be associated with lower Cyclin D1 and CDK4/6 expression, which may suggest an association between ERK1/2 signaling and these G_1_-to-S phase regulatory proteins. ERK1/2 inhibition appeared to increase the G_0_/G_1_ population in both cell lines under Normal pH conditions and in MIA-PaCa-2 cells under Low pH conditions. However, under Low pH conditions, PK-59 cells did not show a comparable increase in the G_0_/G_1_ population despite apparently lower Cyclin D1 and CDK4/6 expression. These observations may be consistent with an association among ERK1/2 inhibition, Cyclin D1/CDK4/6 expression, and cell cycle phase distribution, although the causal relationships among these changes remain uncertain. Rb phosphorylation also appeared to differ between the two cell lines. Whereas phosphorylated Rb did not appear to change markedly in PK-59 cells, it appeared to be lower in MIA-PaCa-2 cells following ERK1/2 inhibition under both pH conditions. These observations may suggest that the relationship between Rb phosphorylation and the G_0_/G_1_ population differs between the two cell lines. In particular, ERK1/2 inhibition under Low pH conditions appeared to be associated with a further increase in the G_0_/G_1_ population only in MIA-PaCa-2 cells. However, the present data do not establish whether the difference in Rb phosphorylation is responsible for this cell line-specific response. The molecular basis for these differences remains unclear. Previous profiling studies have shown that pancreatic cancer cell lines harbor distinct alterations in major tumor suppressor pathways, including CDKN2A, TP53, and SMAD4 (38, 39), which could potentially be associated with differences in cell cycle regulation and responses to ERK1/2 inhibition. However, these possibilities were not directly examined in the present study. p21 expression did not appear to change markedly under the experimental conditions examined, although the present findings do not exclude its involvement in cell cycle regulation. Akt inhibition also appeared to be associated with an increased G_0_/G_1_ population, whereas changes in Cyclin D1, CDK4/6, and Rb phosphorylation were not consistently observed across cell lines and pH conditions. Therefore, the relationship between Akt signaling and the observed changes in cell cycle distribution remains unclear. In contrast, under Low pH conditions, pharmacological Akt inhibition appeared to be accompanied by an increased apoptotic population and changes in apoptosis-related proteins. These observations may indicate that cellular responses to Akt inhibition differ between cell cycle- and apoptosis-related endpoints under acidic conditions; however, they do not establish a causal role for Akt signaling in either response.

Pharmacological Akt inhibition under acidic conditions appeared to be associated with an increased apoptotic population, accompanied by apparent increases in Bad and Bax and a decrease in Bcl-xL. These observations may suggest an association between Akt inhibition and changes in apoptosis-related proteins under acidic conditions; however, they do not establish that Akt signaling is required for the maintenance of cell survival or that the observed changes in Bad, Bax, and Bcl-xL are causally responsible for the apoptotic response. Attenuated ERK1/2 signaling was also accompanied by changes in cell cycle-related parameters and, in some conditions, Bcl-xL expression, although the functional relationships among these observations remain uncertain. Collectively, these findings may be consistent with differential changes in Akt- and ERK1/2-associated signaling under extracellular acidosis, but they do not establish a causal shift toward Akt-mediated survival signaling (**Figure 13**).

**Figure 13:**
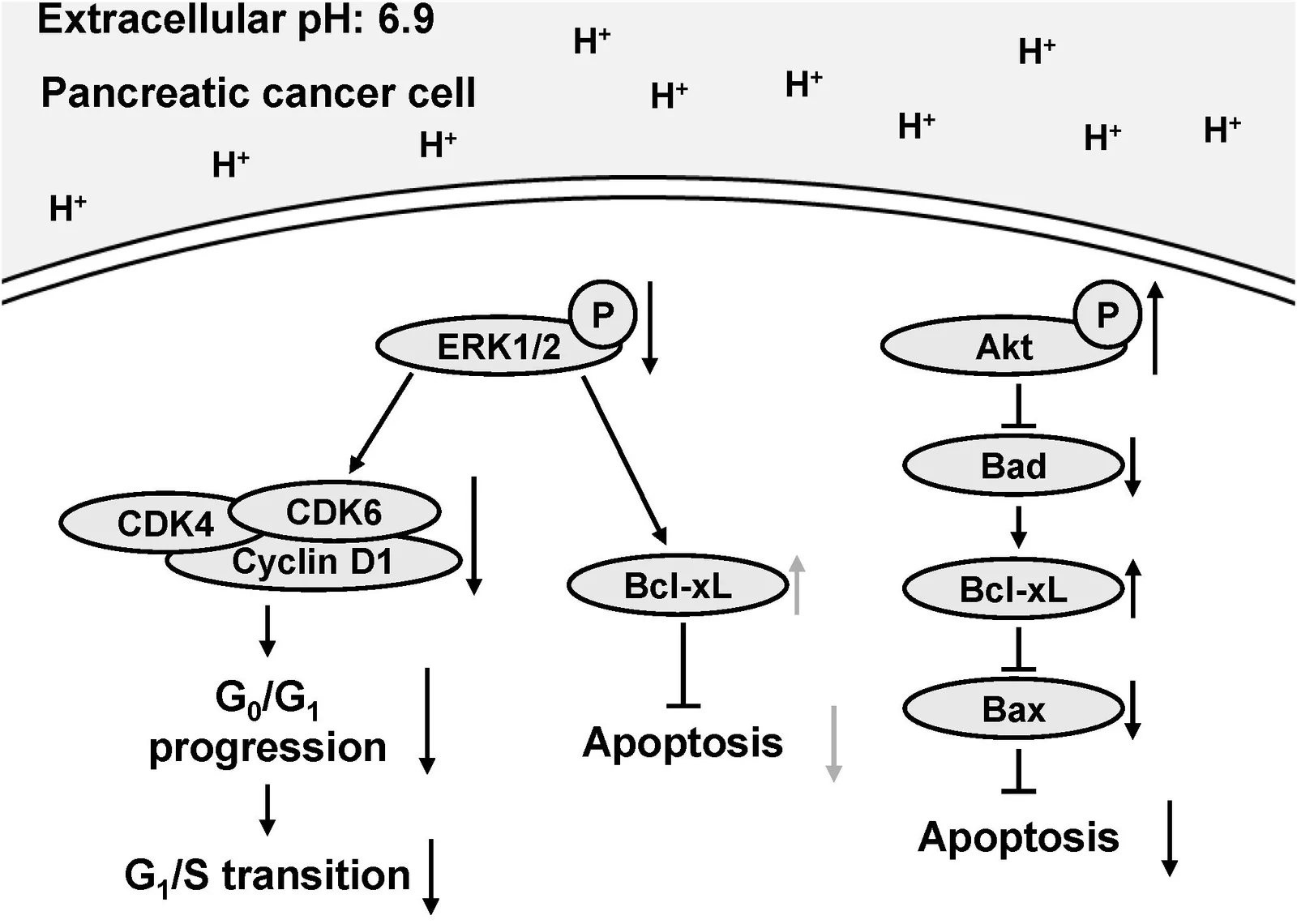
Schematic summary of changes observed under extracellular acidosis. Extracellular acidosis appeared to be associated with reduced proliferation of pancreatic cancer cells, an increased G_0_/G_1_ population, and decreased Cyclin D1-CDK4/6 expression. Apoptotic cell populations did not appear to increase markedly under acidic conditions. Akt phosphorylation appeared to be increased and was accompanied by changes in apoptosis-related proteins, including decreased Bad and Bax and increased Bcl-xL. In contrast, ERK1/2 phosphorylation appeared to be attenuated and was accompanied predominantly by changes in cell cycle-related parameters, while effects on apoptosis appeared to vary between cell lines. Arrows adjacent to each molecule indicate the direction of changes observed under Low pH conditions. Collectively, these observations may be consistent with differential changes in proliferation-associated and survival-associated signaling under extracellular acidosis; however, they do not establish a causal requirement for Akt signaling or a definitive shift toward a survival-dominant state.

The differential changes in ERK1/2 and Akt phosphorylation observed under acidic conditions raise questions regarding the upstream mechanisms associated with these signaling patterns. Canonical receptor tyrosine kinase signaling can regulate both pathways, but the present study did not examine the upstream events responsible for the observed changes in Akt and ERK1/2 phosphorylation. One pathway that may warrant investigation in future studies is proton-sensing G protein-coupled receptor (GPCR) signaling, as extracellular acidosis can be sensed by proton-sensing GPCRs, including GPR4, GPR65, GPR68, and GPR132 (40). GPCR signaling has also been associated with β-arrestin-dependent pathways (23, 41–44), and β-arrestin has been implicated in the regulation of ERK1/2 and Akt signaling. However, the present study did not assess proton-sensing GPCR expression, β-arrestin signaling, or their relationship to the observed Akt and ERK1/2 changes. Therefore, any involvement of proton-sensing GPCRs or β-arrestin-associated signaling in the present findings remains speculative. Future studies directly examining these pathways will be required to determine whether they are associated with the differential changes in Akt and ERK1/2 phosphorylation observed under acidic conditions.

Activation of the PI3K/Akt pathway has been widely implicated in therapeutic resistance (37, 45). In the present study, Akt phosphorylation appeared to be increased under acidic conditions. However, the present experiments did not directly assess therapeutic resistance, and therefore the relationship between the observed increase in Akt phosphorylation and treatment response remains unclear. These observations may indicate that extracellular acidosis is accompanied by alterations in Akt signaling, although the biological significance of this change in pancreatic cancer cell adaptation requires further investigation.

This study has several limitations. First, all experiments were conducted *in vitro* and therefore do not fully recapitulate the complexity of the *in vivo* tumor microenvironment. Second, the experimental model was based solely on extracellular pH and thus represents a simplified condition that does not account for other TME components, including hypoxia, nutrient gradients, stromal interactions, or immune-related factors. Third, although changes in Bad, Bax, and Bcl-xL expression were observed following pharmacological Akt inhibition, the present study did not establish a causal relationship between these changes and the apoptotic response. In addition, mitochondrial membrane potential, metabolic flux, and other functional indices of mitochondrial activity were not examined. Therefore, the functional significance of the observed changes in apoptosis-related proteins, as well as their relationship to Akt signaling and mitochondrial function under acidic conditions, remains uncertain. Furthermore, because the present conclusions are based primarily on pharmacological inhibition, pathway-specific causality cannot be established from these experiments alone. Future studies incorporating complementary genetic approaches, appropriate *in vivo* models, and patient-derived systems will be required to clarify the biological significance and potential translational relevance of these observations.

Future studies will be required to determine whether acidic microenvironments are associated with signaling heterogeneity and phenotypic diversification in pancreatic tumors. The present findings should be considered exploratory and hypothesis-generating. Although altered Akt signaling and an increased apoptotic response to pharmacological Akt inhibition were observed under acidic conditions *in vitro*, these observations do not establish that Akt represents a therapeutic vulnerability or that modulation of Akt signaling would improve treatment responses *in vivo*. Appropriate *in vivo* models, patient-derived systems, and complementary mechanistic approaches will therefore be required to determine the biological and clinical relevance of these findings. More broadly, the present study may provide a framework for investigating how proliferation-associated and survival-associated signaling patterns change under extracellular acidosis, without implying a specific therapeutic strategy at this stage.

Collectively, our findings may suggest that acidic conditions are associated with reduced pancreatic cancer cell proliferation, an increased G_0_/G_1_ population, decreased Cyclin D1/CDK4/6 expression, attenuated ERK1/2 phosphorylation, and increased Akt phosphorylation. The absence of a marked increase in apoptosis under acidic conditions, together with the increased apoptotic response observed following pharmacological Akt inhibition, may indicate an association between extracellular acidosis and altered survival-related cellular responses. However, these observations do not establish a causal requirement for Akt signaling or a definitive shift toward a survival-dominant state. Further mechanistic and *in vivo* studies will be required to clarify the biological significance of these signaling changes.

## 5 Conclusion

Extracellular acidosis appeared to be associated with reduced pancreatic cancer cell proliferation, attenuated ERK1/2 phosphorylation, decreased Cyclin D1-CDK4/6 expression, and an increased G_0_/G_1_ population. Differences in Rb phosphorylation between the two pancreatic cancer cell lines may suggest that the relationship between Rb signaling and cell cycle responses to ERK1/2 inhibition varies between cell lines; however, the present data do not establish a causal role for Rb phosphorylation in these responses. Acidic conditions also appeared to be associated with increased Akt phosphorylation without a marked increase in apoptosis. Pharmacological Akt inhibition under acidic conditions appeared to be accompanied by an increased apoptotic population together with apparent increases in Bad and Bax and a decrease in Bcl-xL, whereas the effects of ERK1/2 inhibition appeared to be more closely associated with changes in cell cycle phase distribution and varied between cell lines with respect to apoptosis. Collectively, these observations may be consistent with differential changes in proliferation-associated and survival-associated signaling under extracellular acidosis; however, they do not establish a causal requirement for Akt signaling or a definitive shift toward a survival-dominant state. These findings are summarized in **Figure 13**.

## Data Availability

The raw data supporting the conclusions of this article will be made available by the authors, without undue reservation.
