# Supplementary material for: Acidic microenvironment promotes pancreatic cancer cell survival via Akt phosphorylation-mediated suppression of apoptosis
Source: Front Oncol. 2026 Sep 22;16:1889708. doi: 10.3389/fonc.2026.1889708 (PMC13638389; doi:10.3389/fonc.2026.1889708)
Supplement: Supplementary file 1 [file SupplementaryFile1.docx]

Supplementary Material

# 1 Supplementary Materials and Methods

## 1.1 Cell lines.

PK-59 cells were cultured in RPMI-1640 with L-glutamine and phenol red (FUJIFILM Wako Pure Chemical Corporation, Osaka, Japan), whereas MIA-PaCa-2 cells were cultured in DMEM (4.5 g/L glucose) with L-glutamine, sodium pyruvate, and 1.5 g/L sodium hydrogen carbonate (Nacalai Tesque, Kyoto, Japan). RPMI-1640 and DMEM media were supplemented with 10% heat-inactivated fetal bovine serum (Thermo Fisher Scientific, Waltham, MA, USA) and 1% penicillin–streptomycin (FUJIFILM Wako Pure Chemical Corporation). Cells were maintained at 37 °C in a humidified incubator with 5% CO₂. No additional cell-line authentication, including short tandem repeat (STR) profiling, was performed in our laboratory. Mycoplasma contamination was routinely assessed using the Venor^®^GeM Classic Mycoplasma Detection Kit (Minerva Biolabs, Berlin, Germany), and all cell cultures tested negative for mycoplasma during the experimental period.

## 1.2 Culture conditions.

RPMI-1640 and DMEM were equilibrated for 24 h at 37 °C under humidified conditions, and pH was measured using a LAQUA act D-72 pH meter (Horiba, Ltd., Kyoto, Japan). Basal pH values were 7.47 for RPMI-1640 and 7.50 for DMEM. Acidic conditions were generated by adding 1 mol/L HNO₃ (Nacalai Tesque) to the basal medium (20 µL/mL for RPMI-1640 and 30 µL/mL for DMEM) and incubating for 24 h at 37 °C. Final pH values were 6.90 for RPMI-1640 and 6.88 for DMEM.

## 1.3 Cell growth assay.

Cells were seeded into Nunc^TM^ Cell-Culture Treated flat-bottom 24-well plates (Thermo Fisher Scientific) and pre-cultured for 24 h. The medium was then replaced with either Normal pH or Low pH medium. Cells were counted every 24 h to 72 h. At 0 h, only viable cells were counted, whereas at 24, 48, and 72 h, both viable and dead cells were counted. The extracellular pH of the culture supernatants was also measured at the 72 h time point using the same pH meter to assess whether the intended pH conditions were maintained at the end of the incubation period.

## 1.4 Cell proliferation assay.

Cells were seeded into Nunc^TM^ Cell-Culture Treated flat-bottom 96-well plates (Thermo Fisher Scientific) and pre-cultured for 24 h. The medium was then replaced with either Normal pH or Low pH medium, and MK-2206 or SCH772984 was applied at 10 serially diluted concentrations for IC_50_ determination. Cells were cultured for 72 h. Absorbance at 450 nm was measured using a microplate reader (GloMax^®^, Promega, Madison, WI, USA).

## 1.5 Phase-contrast imaging of cell morphology.

Cells were seeded into Nunc^TM^ Cell-Culture Treated flat-bottom 6-well plates (Thermo Fisher Scientific) and pre-cultured for 24 h. The medium was then replaced with either Normal pH or Low pH medium, and the cells were cultured for 72 h. To examine the effects of Akt or ERK1/2 inhibition on cell morphology, cells were seeded and pre-cultured for 24 h, followed by replacement with Normal pH or Low pH medium and culture for 72 h. For inhibitor studies, MK-2206 and SCH772984 were applied under both Normal pH and Low pH conditions. MK-2206 was used at the IC_50_ concentration determined under Normal pH conditions for each cell line (20 µM for PK-59 cells and 15 µM for MIA-PaCa-2 cells), whereas SCH772984 was used at the IC_50_ concentration determined under Normal pH conditions for each cell line (35 µM for PK-59 cells and 8 nM for MIA-PaCa-2 cells). The cells were then cultured for 72 h. After 72 h of treatment, cellular morphology was examined using an IX70 inverted phase-contrast microscope equipped with a TH4-100 illumination system (Olympus, Tokyo, Japan). Images were captured using an MC120 HD digital camera (Leica Microsystems, Wetzlar, Germany), and representative fields were selected for each experimental condition.

## 1.6 Cell cycle analysis.

To examine the effects of Low pH conditions on the cell cycle, cells were seeded and pre-cultured for 24 h. The medium was then replaced with either Normal pH or Low pH medium, and the cells were cultured for 72 h. To examine the effects of Akt or ERK1/2 inhibition on the cell cycle, cells were seeded and pre-cultured for 24 h, followed by replacement with Normal pH or Low pH medium and culture for 72 h. For inhibitor studies, MK-2206 and SCH772984 were applied under both Normal pH and Low pH conditions. MK-2206 was used at the IC_50_ concentration determined under Normal pH conditions for each cell line (20 µM for PK-59 cells and 15 µM for MIA-PaCa-2 cells), whereas SCH772984 was used at the IC_50_ concentration determined under Normal pH conditions for each cell line (35 µM for PK-59 cells and 8 nM for MIA-PaCa-2 cells). After 72 h of treatment, cells were collected, washed with phosphate-buffered saline (PBS) (-), and suspended in 70% ethanol (FUJIFILM Wako Pure Chemical Corporation) that had been pre-cooled to -30 °C. They were then fixed by incubating at -30 °C for at least 12 h. Following fixation, the 70% ethanol was removed, and cells were stained at 4 °C for 30 min with PBS (-) containing 20 µg/mL propidium iodide (PI; FUJIFILM Wako Pure Chemical Corporation) and 100 µg/mL RNase (Sigma Aldrich, St. Louis, MO, USA). Stained cells were analyzed using the BD LSRFortessa^TM^ (BD Biosciences, San Jose, CA, USA) flow cytometer equipped with the BD FACSDiva^TM^ software (BD Biosciences). The results were analyzed using the FlowJo version 10.10.0 software (FlowJo LLC, Ashland, OR, USA). Each dataset represents an independently acquired FCS file, and all histogram and dot plot representations were generated from corresponding matched datasets without reuse across conditions or replicates. Gating was performed consistently using predefined criteria across all samples. Biological samples were collected on separate experimental days; however, all samples were processed and analyzed under identical flow cytometry acquisition settings. Raw data traceability was maintained throughout, ensuring one-to-one correspondence between displayed plots and original FCS files.

## 1.7 Apoptosis analysis.

To examine the effects of Low pH conditions on apoptosis, cells were seeded and pre-cultured for 24 h. The medium was then replaced with either Normal pH or Low pH medium, and the cells were cultured for 72 h. To examine the effects of Akt or ERK1/2 inhibition on apoptosis, cells were seeded and pre-cultured for 24 h, followed by replacement with Normal pH or Low pH medium and culture for 72 h. For inhibitor studies, MK-2206 and SCH772984 were applied under both Normal pH and Low pH conditions. MK-2206 was used at the IC_50_ concentration determined under Normal pH conditions for each cell line (20 µM for PK-59 cells and 15 µM for MIA-PaCa-2 cells), whereas SCH772984 was used at the IC_50_ concentration determined under Normal pH conditions for each cell line (35 µM for PK-59 cells and 8 nM for MIA-PaCa-2 cells). After 72 h of treatment, cells were collected, washed with PBS (-), and suspended in the Binding Buffer included with the eBioscience^TM^Annexin V FITC Apoptosis Detection Kit (Thermo Fisher Scientific). Cells were stained with Annexin V-FITC and PI. Annexin V (+)/PI (-) cells were defined as the early apoptotic cells, whereas Annexin V (+)/PI (+) cells were defined as the late apoptotic cells. Stained cells were analyzed using the BD LSRFortessa^TM^ flow cytometer equipped with the BD FACSDiva^TM^ software. The results were analyzed using the FlowJo version 10.10.0 software. Each dataset represents an independently acquired FCS file, and all dot plot representations were generated from corresponding matched datasets without reuse across conditions or replicates. Gating was performed consistently using predefined criteria across all samples. Biological samples were collected on separate experimental days; however, all samples were processed and analyzed on the same day under identical flow cytometry acquisition settings to minimize technical variability. Raw data traceability was maintained throughout, ensuring one-to-one correspondence between displayed plots and original FCS files.

## 1.8 Western blotting.

To examine the effects of Low pH conditions on protein expression, cells were seeded and pre-cultured for 24 h. The medium was then replaced with either Normal pH or Low pH medium, and the cells were cultured for 72 h. To examine the effects of Akt or ERK1/2 inhibition on protein expression, cells were seeded and pre-cultured for 24 h, followed by replacement with Normal pH or Low pH medium and culture for 72 h. For inhibitor studies, MK-2206 and SCH772984 were applied under both Normal pH and Low pH conditions. MK-2206 was used at the IC_50_ concentration determined under Normal pH conditions for each cell line (20 µM for PK-59 cells and 15 µM for MIA-PaCa-2 cells), whereas SCH772984 was used at the IC_50_ concentration determined under Normal pH conditions for each cell line (35 µM for PK-59 cells and 8 nM for MIA-PaCa-2 cells). After 24 h or 72 h of treatment depending on the experimental design, cells were collected, washed with PBS (-), and lysed by sonication on ice in RIPA Buffer (FUJIFILM Wako Pure Chemical Corporation) containing a protease inhibitor cocktail (Sigma Aldrich), 40 mM sodium fluoride (FUJIFILM Wako Pure Chemical Corporation), and 2 mM sodium orthovanadate (Sigma Aldrich). Lysates were centrifuged at 14,800 rpm at 4 °C for 20 min, and the supernatants were collected as protein extracts. After that, protein concentrations were determined using the PierceTM BCA Protein Assay Kit (Thermo Fisher Scientific). Samples were mixed with 3-Mercapto-1,2-propanediol containing sample buffer (FUJIFILM Wako Pure Chemical Corporation) and heated at 98 °C for 5 min. Biological samples were collected across independent experimental replicates on different days. For each replicate, protein samples corresponding to all experimental conditions were processed in parallel and subjected to sodium dodecyl sulfate-polyacrylamide gel electrophoresis (SDS-PAGE), transferred onto polyvinylidene difluoride (PVDF) membranes (Merck Millipore, Burlington, MA, USA), and immunodetected on the same day under identical experimental conditions to minimize technical variability and ensure direct comparability across conditions. All comparisons were performed using samples processed in parallel within the same experimental run. Equal amounts of protein were subjected to SDS-PAGE and transferred onto PVDF membranes. Precision Plus Protein Prestained Standards (Bio-Rad Laboratories, CA, USA) or ExcelBand 3-color Regular Range Protein Marker (SMOBIO Technology, Inc., Hsinchu, Taiwan) were used as molecular weight markers. Membranes were blocked with Blocking One-P (Nacalai Tesque), 1-5% skim milk (Megmilk Snow Brand Co., Ltd., Tokyo, Japan), or 1-2.5% bovine serum albumin (BSA; FUJIFILM Wako Pure Chemical Corporation) dissolved in Tris-buffered saline containing Tween-20 (TBS-T), depending on the antibody. Blocking One-P was applied for 20 min at room temperature, whereas skim milk and BSA were applied for 30 min to 1 h. Membranes were incubated with primary antibodies overnight at 4 °C, followed by incubation with horseradish peroxidase (HRP)-conjugated anti-rabbit or anti-mouse IgG secondary antibodies (R&D Systems, Minneapolis, MN, USA) for 1 h at room temperature. Antibodies were diluted in TBS-T or Can Get Signal^®^ Immunoreaction Enhancer Solution (TOYOBO CO., LTD., Osaka, Japan). Protein bands were detected using the ECL Prime Western Blotting Detection Kits (Cytiva, Tokyo, Japan) and visualized with LuminoGraph I (ATTO corporation, Tokyo, Japan) equipped with ImageSaver6 software (ATTO corporation). Band intensities were quantified using CSAnalyzer4 (ATTO corporation). β-actin was used as an internal control. Primary antibodies used in this study were purchased from BD Biosciences, Cell Signaling Technology (Beverly, MA, USA), Santa Cruz Biotechnology (Dallas, TX, USA), or Sigma Aldrich, and are listed in Supplementary Table S1.

# 2 Supplementary Figures and Tables

## 2.1 Supplementary Figures

**Supplementary Figure S1. Effects of acidic conditions on cell morphology.**

PK-59 and MIA-PaCa-2 cells were cultured for 72 h under Normal pH or Low pH conditions. (A and E) Representative phase-contrast images of PK-59 and MIA-PaCa-2 cells cultured under Normal pH at 4× magnification. (B and F) Representative phase-contrast images of PK-59 and MIA-PaCa-2 cells cultured under Low pH at 4× magnification. (C and G) Representative phase-contrast images of PK-59 and MIA-PaCa-2 cells cultured under Normal pH at 10× magnification. (D and H) Representative phase-contrast images of PK-59 and MIA-PaCa-2 cells cultured under Low pH at 10× magnification. Scale bars, 250 μm.

**Supplementary Figure S2.** **Effects of acidic conditions on cell cycle phase distribution and apoptosis.**

PK-59 and MIA-PaCa-2 cells were cultured for 72 h under Normal pH or Low pH conditions. (A) Representative histograms of cell cycle phase distribution in PK-59 cells. (B) Representative histograms of cell cycle phase distribution in MIA-PaCa-2 cells. (C) Representative Annexin V/PI flow cytometry plots of PK-59 cells. (D) Representative Annexin V/PI flow cytometry plots of MIA-PaCa-2 cells. Representative histograms and flow cytometry plots from three independent experiments are shown.

**Supplementary Figure S3. Effects of acidic conditions on the expression levels of proteins regulating the G_1_-to-S phase transition.**

PK-59 and MIA-PaCa-2 cells were cultured for 72 h under Normal pH or Low pH conditions. (A and F) Quantification of Cyclin D1 expression in PK-59 and MIA-PaCa-2 cells, respectively. (B and G) Quantification of CDK4 expression in PK-59 and MIA-PaCa-2 cells, respectively. (C and H) Quantification of CDK6 expression in PK-59 and MIA-PaCa-2 cells, respectively. (D and I) Quantification of phospho-Rb normalized to total Rb in PK-59 and MIA-PaCa-2 cells, respectively. (E and J) Quantification of p21 expression in PK-59 and MIA-PaCa-2 cells, respectively. Cyclin D1, CDK4, CDK6, and p21 expression levels were normalized to the corresponding β-actin from the same membrane. Quantitative data represent the mean ± SD from three independent experiments, each performed in triplicate. Statistical comparisons were performed using an unpaired Student’s t-test. *p < 0.05; **p < 0.01; ***p < 0.001.

**Supplementary Figure S4. Effects of acidic conditions on the expression levels of proteins regulating apoptosis.**

PK-59 and MIA-PaCa-2 cells were cultured for 72 h under Normal pH or Low pH conditions. (A and D) Quantification of Bad expression in PK-59 and MIA-PaCa-2 cells, respectively. (B and E) Quantification of Bax expression in PK-59 and MIA-PaCa-2 cells, respectively. (C and F) Quantification of Bcl-xL expression in PK-59 and MIA-PaCa-2 cells, respectively. All target proteins were normalized to their corresponding β-actin derived from the same membrane. Quantitative data represent the mean ± SD from three independent experiments, each performed in triplicate. Statistical comparisons were performed using an unpaired Student’s t-test. *p < 0.05; **p < 0.01; ***p < 0.001.

**Supplementary Figure S5. Effects of acidic conditions on Akt and ERK1/2 phosphorylation.**

PK-59 and MIA-PaCa-2 cells were cultured for 72 h under Normal pH or Low pH conditions. (A and B) Quantification of phospho-Akt normalized to total Akt in PK-59 and MIA-PaCa-2 cells, respectively. (C and D) Quantification of phospho-ERK1/2 normalized to total ERK1/2 in PK-59 and MIA-PaCa-2 cells, respectively. Quantitative data represent the mean ± SD from three independent experiments, each performed in triplicate. Statistical comparisons were performed using an unpaired Student’s t-test. *p < 0.05; **p < 0.01; ***p < 0.001.

**Supplementary Figure S6. Effects of Akt inhibition on Akt phosphorylation.**

PK-59 and MIA-PaCa-2 cells were cultured for 24 h with or without MK-2206 under Normal pH or Low pH conditions. (A and C) Representative cropped immunoblot images of phospho-Akt, total Akt, and β-actin in PK-59 and MIA-PaCa-2 cells, respectively. (B and D) Quantification of phospho-Akt normalized to total Akt in PK-59 and MIA-PaCa-2 cells, respectively. All immunoblot images are representative of three independent experiments. Quantitative data represent the mean ± SD from three independent experiments, each performed in triplicate. Comparisons among multiple groups were performed using one-way ANOVA, followed by Bonferroni’s multiple comparisons test. **p < 0.01; ***p < 0.001; ns, not significant.

**Supplementary Figure S7. Effects of Akt inhibition on cell morphology.**

PK-59 and MIA-PaCa-2 cells were cultured for 72 h with or without MK-2206 under Normal pH or Low pH conditions. (A and I) Representative phase-contrast images of PK-59 and MIA-PaCa-2 cells cultured under Normal pH without MK-2206 at 4× magnification. (B and J) Representative phase-contrast images of PK-59 and MIA-PaCa-2 cells cultured under Low pH without MK-2206 at 4× magnification. (C and K) Representative phase-contrast images of PK-59 and MIA-PaCa-2 cells cultured under Normal pH with MK-2206 at 4× magnification. (D and L) Representative phase-contrast images of PK-59 and MIA-PaCa-2 cells cultured under Low pH with MK-2206 at 4× magnification. (E and M) Representative phase-contrast images of PK-59 and MIA-PaCa-2 cells cultured under Normal pH without MK-2206 at 10× magnification. (F and N) Representative phase-contrast images of PK-59 and MIA-PaCa-2 cells cultured under Low pH without MK-2206 at 10× magnification. (G and O) Representative phase-contrast images of PK-59 and MIA-PaCa-2 cells cultured under Normal pH with MK-2206 at 10× magnification. (H and P) Representative phase-contrast images of PK-59 and MIA-PaCa-2 cells cultured under Low pH with MK-2206 at 10× magnification. Scale bars, 250 μm.

**Supplementary Figure S8. Effects of Akt inhibition on cell cycle phase distribution.**

Cell cycle analysis of PK-59 and MIA-PaCa-2 cells cultured for 72 h with or without MK-2206 under Normal pH or Low pH conditions. (A) Representative histograms of cell cycle phase distribution in PK-59 cells. (B) Representative histograms of cell cycle phase distribution in MIA-PaCa-2 cells. (A and B) Representative histograms from three independent experiments are shown.

**Supplementary Figure S9. Effects of Akt inhibition on the expression levels of proteins regulating the G_1_-to-S phase transition.**

PK-59 and MIA-PaCa-2 cells were cultured for 24 h with or without MK-2206 under Normal pH or Low pH conditions. (A and F) Quantification of Cyclin D1 expression in PK-59 and MIA-PaCa-2 cells, respectively. (B and G) Quantification of CDK4 expression in PK-59 and MIA-PaCa-2 cells, respectively. (C and H) Quantification of CDK6 expression in PK-59 and MIA-PaCa-2 cells, respectively. (D and I) Quantification of phospho-Rb normalized to total Rb in PK-59 and MIA-PaCa-2 cells, respectively. (E and J) Quantification of p21 expression in PK-59 and MIA-PaCa-2 cells, respectively. Cyclin D1, CDK4, CDK6, and p21 expression levels were normalized to the corresponding β-actin from the same membrane. Quantitative data represent the mean ± SD from three independent experiments, each performed in triplicate. Statistical comparisons among multiple groups were performed using one-way ANOVA, followed by Bonferroni’s multiple comparisons test. *p < 0.05; **p < 0.01; ***p < 0.001; ns, not significant.

**Supplementary Figure S10. Effects of Akt inhibition on apoptosis and the expression levels of proteins regulating apoptosis.**

(A and B) Apoptosis analysis of PK-59 and MIA-PaCa-2 cells cultured for 72 h with or without MK-2206 under Normal pH or Low pH conditions. (A) Representative Annexin V/PI flow cytometry plots of PK-59 cells. (B) Representative Annexin V/PI flow cytometry plots of MIA-PaCa-2 cells. (A and B) Representative Annexin V/PI flow cytometry plots from three independent experiments are shown. (C-H) Quantification of Bad, Bax, and Bcl-xL expression in PK-59 and MIA-PaCa-2 cells cultured for 24 h with or without MK-2206 under Normal pH or Low pH conditions. (C and F) Quantification of Bad expression in PK-59 and MIA-PaCa-2 cells, respectively. (D and G) Quantification of Bax expression in PK-59 and MIA-PaCa-2 cells, respectively. (E and H) Quantification of Bcl-xL expression in PK-59 and MIA-PaCa-2 cells, respectively. All target proteins were normalized to their corresponding β-actin derived from the same membrane. Quantitative data represent the mean ± SD from three independent experiments, each performed in triplicate. Statistical comparisons among multiple groups were performed using one-way ANOVA, followed by Bonferroni’s multiple comparisons test. ***p < 0.001; ns, not significant.

**Supplementary Figure S11. Effects of ERK1/2 inhibition on ERK1/2 phosphorylation.**

PK-59 and MIA-PaCa-2 cells were cultured for 24 h with or without SCH772984 under Normal pH or Low pH conditions. (A and C) Representative cropped immunoblot images of phospho-ERK1/2, total ERK1/2, and β-actin in PK-59 and MIA-PaCa-2 cells, respectively. (B and D) Quantification of phospho-ERK1/2 normalized to total ERK1/2 in PK-59 and MIA-PaCa-2 cells, respectively. All immunoblot images are representative of three independent experiments. Quantitative data represent the mean ± SD from three independent experiments, each performed in triplicate. Comparisons among multiple groups were performed using one-way ANOVA, followed by Bonferroni’s multiple comparisons test. *p < 0.05; ***p < 0.001; ns, not significant.

**Supplementary Figure S12. Effects of ERK1/2 inhibition on cell morphology.**

PK-59 and MIA-PaCa-2 cells were cultured for 72 h with or without SCH772984 under Normal pH or Low pH conditions. (A and I) Representative phase-contrast images of PK-59 and MIA-PaCa-2 cells cultured under Normal pH without SCH772984 at 4× magnification. (B and J) Representative phase-contrast images of PK-59 and MIA-PaCa-2 cells cultured under Low pH without SCH772984 at 4× magnification. (C and K) Representative phase-contrast images of PK-59 and MIA-PaCa-2 cells cultured under Normal pH with SCH772984 at 4× magnification. (D and L) Representative phase-contrast images of PK-59 and MIA-PaCa-2 cells cultured under Low pH with SCH772984 at 4× magnification. (E and M) Representative phase-contrast images of PK-59 and MIA-PaCa-2 cells cultured under Normal pH without SCH772984 at 10× magnification. (F and N) Representative phase-contrast images of PK-59 and MIA-PaCa-2 cells cultured under Low pH without SCH772984 at 10× magnification. (G and O) Representative phase-contrast images of PK-59 and MIA-PaCa-2 cells cultured under Normal pH with SCH772984 at 10× magnification. (H and P) Representative phase-contrast images of PK-59 and MIA-PaCa-2 cells cultured under Low pH with SCH772984 at 10× magnification. Scale bars, 250 μm.

**Supplementary Figure S13. Effects of ERK1/2 inhibition on cell cycle phase distribution.**

Cell cycle analysis of PK-59 and MIA-PaCa-2 cells cultured for 72 h with or without SCH772984 under Normal pH or Low pH conditions. (A) Representative histograms of cell cycle phase distribution in PK-59 cells. (B) Representative histograms of cell cycle phase distribution in MIA-PaCa-2 cells. (A and B) Representative histograms from three independent experiments are shown.

**Supplementary Figure S14. Effects of ERK1/2 inhibition on the expression levels of proteins regulating the G_1_-to-S phase transition.**

PK-59 and MIA-PaCa-2 cells were cultured for 24 h with or without SCH772984 under Normal pH or Low pH conditions. (A and F) Quantification of Cyclin D1 expression in PK-59 and MIA-PaCa-2 cells, respectively. (B and G) Quantification of CDK4 expression in PK-59 and MIA-PaCa-2 cells, respectively. (C and H) Quantification of CDK6 expression in PK-59 and MIA-PaCa-2 cells, respectively. (D and I) Quantification of phospho-Rb normalized to total Rb in PK-59 and MIA-PaCa-2 cells, respectively. (E and J) Quantification of p21 expression in PK-59 and MIA-PaCa-2 cells, respectively. Cyclin D1, CDK4, CDK6, and p21 expression levels were normalized to the corresponding β-actin from the same membrane. Quantitative data represent the mean ± SD from three independent experiments, each performed in triplicate. Statistical comparisons among multiple groups were performed using one-way ANOVA, followed by Bonferroni’s multiple comparisons test. *p < 0.05; **p < 0.01; ***p < 0.001; ns, not significant.

**Supplementary Figure S15. Effects of ERK1/2 inhibition on apoptosis and the expression levels of proteins regulating apoptosis.**

(A and B) Apoptosis analysis of PK-59 and MIA-PaCa-2 cells cultured for 72 h with or without SCH772984 under Normal pH or Low pH conditions. (A) Representative Annexin V/PI flow cytometry plots of PK-59 cells. (B) Representative Annexin V/PI flow cytometry plots of MIA-PaCa-2 cells. (A and B) Representative Annexin V/PI flow cytometry plots from three independent experiments are shown. (C-H) Quantification of Bad, Bax, and Bcl-xL expression in PK-59 and MIA-PaCa-2 cells cultured for 24 h with or without SCH772984 under Normal pH or Low pH conditions. (C and F) Quantification of Bad expression in PK-59 and MIA-PaCa-2 cells, respectively. (D and G) Quantification of Bax expression in PK-59 and MIA-PaCa-2 cells, respectively. (E and H) Quantification of Bcl-xL expression in PK-59 and MIA-PaCa-2 cells, respectively. All target proteins were normalized to their corresponding β-actin derived from the same membrane. Quantitative data represent the mean ± SD from three independent experiments, each performed in triplicate. Statistical comparisons among multiple groups were performed using one-way ANOVA, followed by Bonferroni’s multiple comparisons test. *p < 0.05; **p < 0.01; ***p < 0.001; ns, not significant.

## 2.2 Supplementary Tables

**Supplementary Table S1. Primary antibodies used for western blotting in this study.**

| Primary antibody | Source | Identifier | Dilution |
| --- | --- | --- | --- |
| Cyclin D1 | BD Biosciences | 554180 | 1:500 |
| CDK4 (DCS-35) | Santa Cruz Biotechnology | sc-56277  sc-23896 | 1:200 |
| CDK6 (B-10) | Santa Cruz Biotechnology | sc-7961 | 1:500 |
| Phospho-Rb  (Ser807/811) (D20B12) | Cell Signaling Technology | #8516 | 1:1000 |
| Rb (D20) | Cell Signaling Technology | #9313 | 1:1000 |
| p21 (C-19) | Santa Cruz Biotechnology | sc-397 | 1:200 |
| Bad | BD Biosciences | 610391 | 1:200 |
| Bax (N-20) | Santa Cruz Biotechnology | sc-493 | 1:100 |
| Bcl-xL | Cell Signaling Technology | #2762 | 1:500 |
| Phospho-Akt (Ser473) | Cell Signaling Technology | #9271 | 1:1000 |
| Akt | Cell Signaling Technology | #9272 | 1:1000 |
| Phospho-p44/42 MAPK (Erk1/2)  (Thr202/Tyr204) (E10) | Cell Signaling Technology | #9106 | 1:1000 |
| p44/42 MAPK (Erk1/2) | Cell Signaling Technology | #9102 | 1:1000 |
| β-actin | Sigma Aldrich | A5441 | 1:5000 |

Primary antibodies used for western blotting are listed, including antibody name, source, identifier, and working dilution.
